# Supplementary material for: Palmitoyltransferase ZDHHC6 promotes colon tumorigenesis by targeting PPARγ-driven lipid biosynthesis via regulating lipidome metabolic reprogramming
Source: J Exp Clin Cancer Res. 2024 Aug 16;43:227. doi: 10.1186/s13046-024-03154-0 (PMC11328492; doi:10.1186/s13046-024-03154-0)
Supplement: Supplementary file 1 — Supplementary Material 1 [file 13046_2024_3154_MOESM1_ESM.docx]

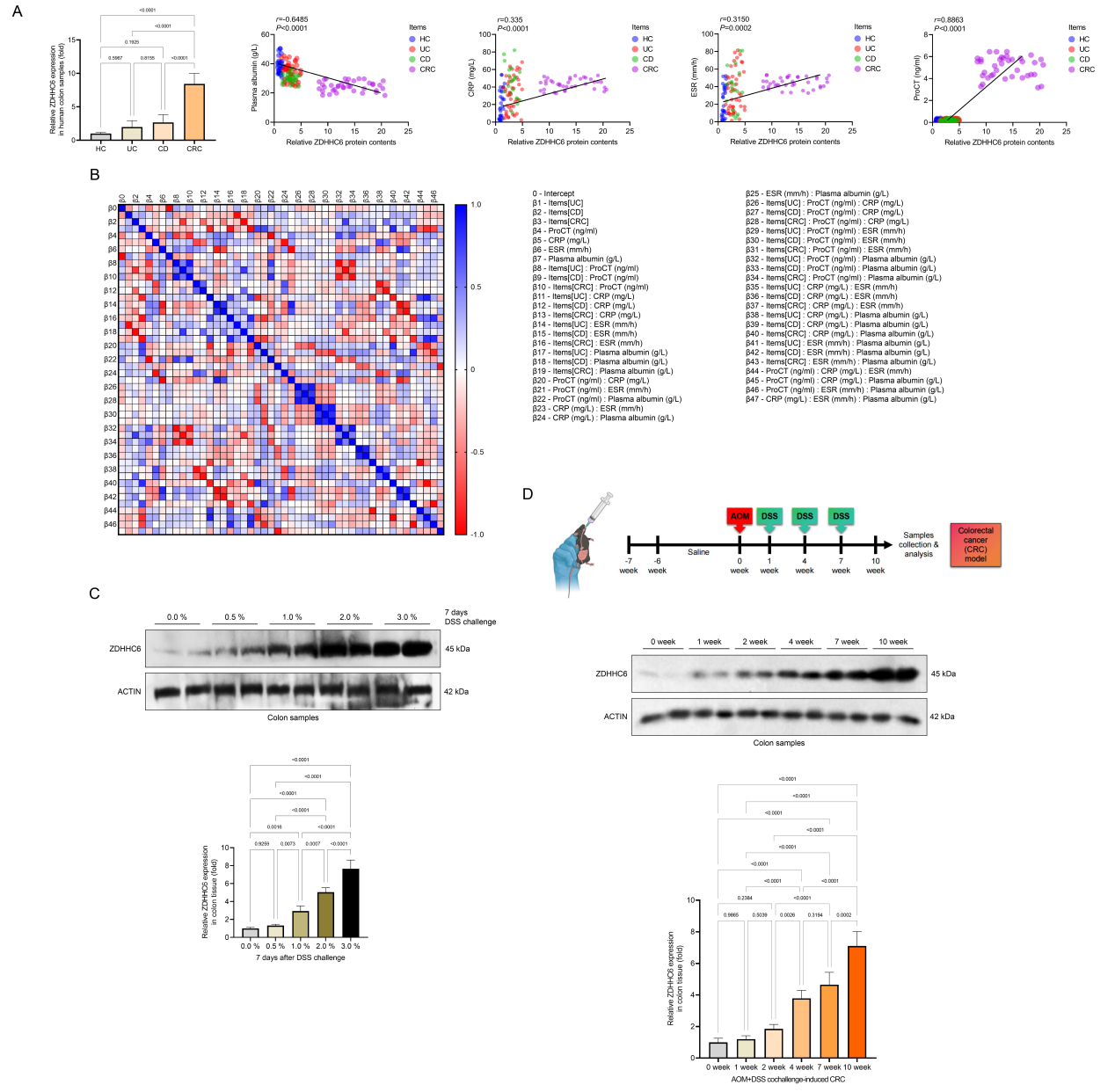


**Supplementary figure 1.** **ZDHHC6 expression is upregulated in IBD patients and mouse colitis-related CRC model.**

(**A**) Left, the ZDHHC6 protein expression was analyzed in 30 healthy control (HC) individuals and 40 patients with Crohn's disease (CD), 45 with ulcerative colitis (UC), and 62 with colorectal cancer (CRC). Right, the Pearson correlation analysis was conducted to assess the connection between colon ZDHHC6 protein expression levels and ProCT, CRP, ESR, and plasma ALB levels in HC, CD, UC, and CRC groups. *n*=177 per parameter.
(**B**) Pearson correlation coefficient (r) was used in a multiple linear regression analysis to show the overall relationship between ZDHHC6 expression in human colon samples and specified parameter indices. *n*=177 per parameter.
(**C**) The DSS dilution was given to wild-type C57BL/6 mice. Colon tissue was collected on day 7. ZDHHC6 protein expression was assessed by western blotting assay in specified groups. *n* = 5 per group.
(**D**) Experimental design involving arabinose pre-treatment, followed by induction of colitis-associated cancer in mice using azoxymethane/dextran sulphate sodium (AOM/DSS). Western blot experiment displaying ZDHHC6 protein expression in colon samples throughout time. *n* = 5 per group.

Data are expressed as mean ± SEM. The relevant experiments presented in this part were performed independently at least three times. *P* <0.05 indicates statistical significance.
