# Supplementary material for: Palmitoyltransferase ZDHHC6 promotes colon tumorigenesis by targeting PPARγ-driven lipid biosynthesis via regulating lipidome metabolic reprogramming
Source: J Exp Clin Cancer Res. 2024 Aug 16;43:227. doi: 10.1186/s13046-024-03154-0 (PMC11328492; doi:10.1186/s13046-024-03154-0)
Supplement: Supplementary file 2 — Supplementary Material 2 [file 13046_2024_3154_MOESM2_ESM.docx]

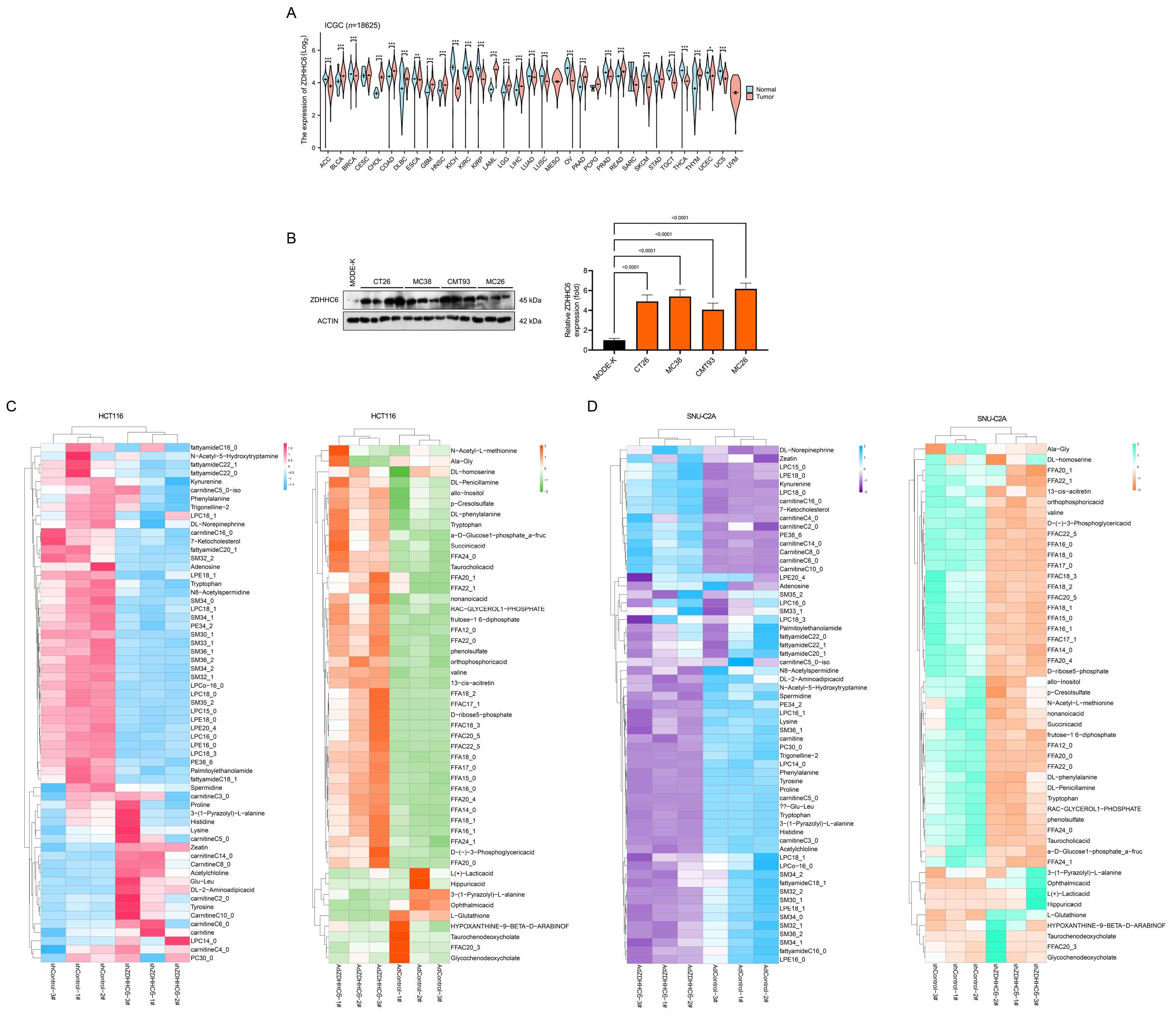


**Supplementary figure 2. ZDHHC6 is abundantly present in colorectal cancer cells and enhances the accumulation of lipids and lipid-like molecules.**

(**A**) Analyzing pancarcinoma utilizing ICGC datasets to quantify ZDHHC6 expression levels across different types of cancers.
(**B**) Western blotting bands displaying ZDHHC6 expression in mouse colon epithelial cell line (MODE-K) and other mice CRC cell lines (CT26, MC38, CMT93, and MC26). *P* < 0.05 compared to MODE-K group; *n* = 5 per group.
(**C**) Heatmap study of altered metabolites in HCT116 cells transfected with shZDHHC6 or overexpressed with Ad*ZDHHC6*. Untargeted metabolomic analysis was performed using LC-MS, and the data was normalized based on the overall peak area. *P* value is less than 0.05, determined using an unpaired two-tailed Student's *t*-test. The range from -2.0 to 2.0 represents the fold change (Fc).
(**D**) Heatmap study of altered metabolites in SNU-C2A cells transfected with shZDHHC6 or overexpressed with Ad*ZDHHC6*. Untargeted metabolomic analysis was conducted using LC-MS, and the data were normalized based on the overall peak area. The *P* value is less than 0.05, indicating statistical significance in an unpaired two-tailed Student's *t*-test. The range from -2.0 to 2.0 represents the fold change (Fc).

Data are expressed as mean ± SEM. The relevant experiments presented in this part were performed independently at least three times. *P* <0.05 indicates statistical significance.
