# Supplementary material for: Palmitoyltransferase ZDHHC6 promotes colon tumorigenesis by targeting PPARγ-driven lipid biosynthesis via regulating lipidome metabolic reprogramming
Source: J Exp Clin Cancer Res. 2024 Aug 16;43:227. doi: 10.1186/s13046-024-03154-0 (PMC11328492; doi:10.1186/s13046-024-03154-0)
Supplement: Supplementary file 3 — Supplementary Material 3 [file 13046_2024_3154_MOESM3_ESM.docx]

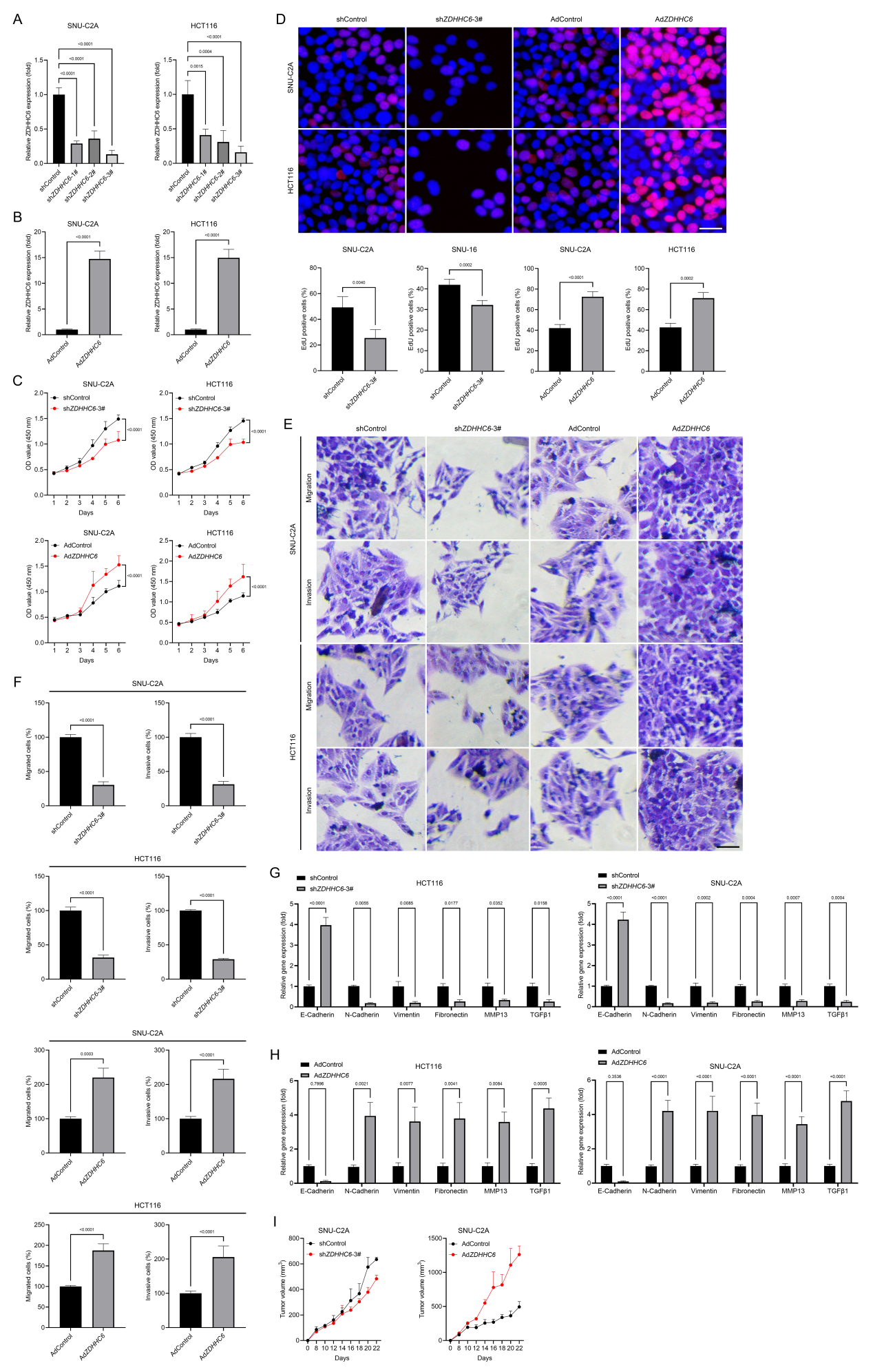


**Supplementary figure 3. ZDHHC6 promotes the cell proliferation of CRC cells.**

(**A**) Quantitative PCR investigation demonstrated the mRNA expression levels of ZDHHC6 in colorectal cancer cell lines post transfection. There are 5 individuals in each group.
(**B**) Quantitative PCR analysis was conducted to measure the mRNA expression levels of ZDHHC6 in colorectal cancer cell lines following transfection with Ad*ZDHHC6*. There are 5 individuals in each group.
(**C**) CCK-8 experiments were conducted to evaluate the impact of ZDHHC6 on colorectal cancer cell growth. There are 5 individuals in each group.
(**D**) EdU experiments were conducted to evaluate the impact of ZDHHC6 on colorectal cancer cell growth. *n* = 5 per group. Scale bars, 20 μm.
(**E, F**) Transwell assay was conducted to assess cell migration and invasion in SNU-C2A and HCT116 cells following transfection with shZDHHC6 or ZDHHC6 overexpression. There are 5 individuals in each group. Scale bars, 100 μm.
(**G, H**) EMT-associated genes in SNU-C2A and HCT116 cells were analyzed using qPCR after manipulating ZDHHC6 expression through knockdown or over-expression transfection. There are 5 individuals in each group. Scale bars, 100 μm.
(**I**) SNU-C2A tumor growth over time following transduction with shZDHHC6 or Ad*ZDHHC6*. There are 10 individuals in each group.

Data are expressed as mean ± SEM. The relevant experiments presented in this part were performed independently at least three times. *P* <0.05 indicates statistical significance.
