# Supplementary material for: Palmitoyltransferase ZDHHC6 promotes colon tumorigenesis by targeting PPARγ-driven lipid biosynthesis via regulating lipidome metabolic reprogramming
Source: J Exp Clin Cancer Res. 2024 Aug 16;43:227. doi: 10.1186/s13046-024-03154-0 (PMC11328492; doi:10.1186/s13046-024-03154-0)
Supplement: Supplementary file 4 — Supplementary Material 4 [file 13046_2024_3154_MOESM4_ESM.docx]

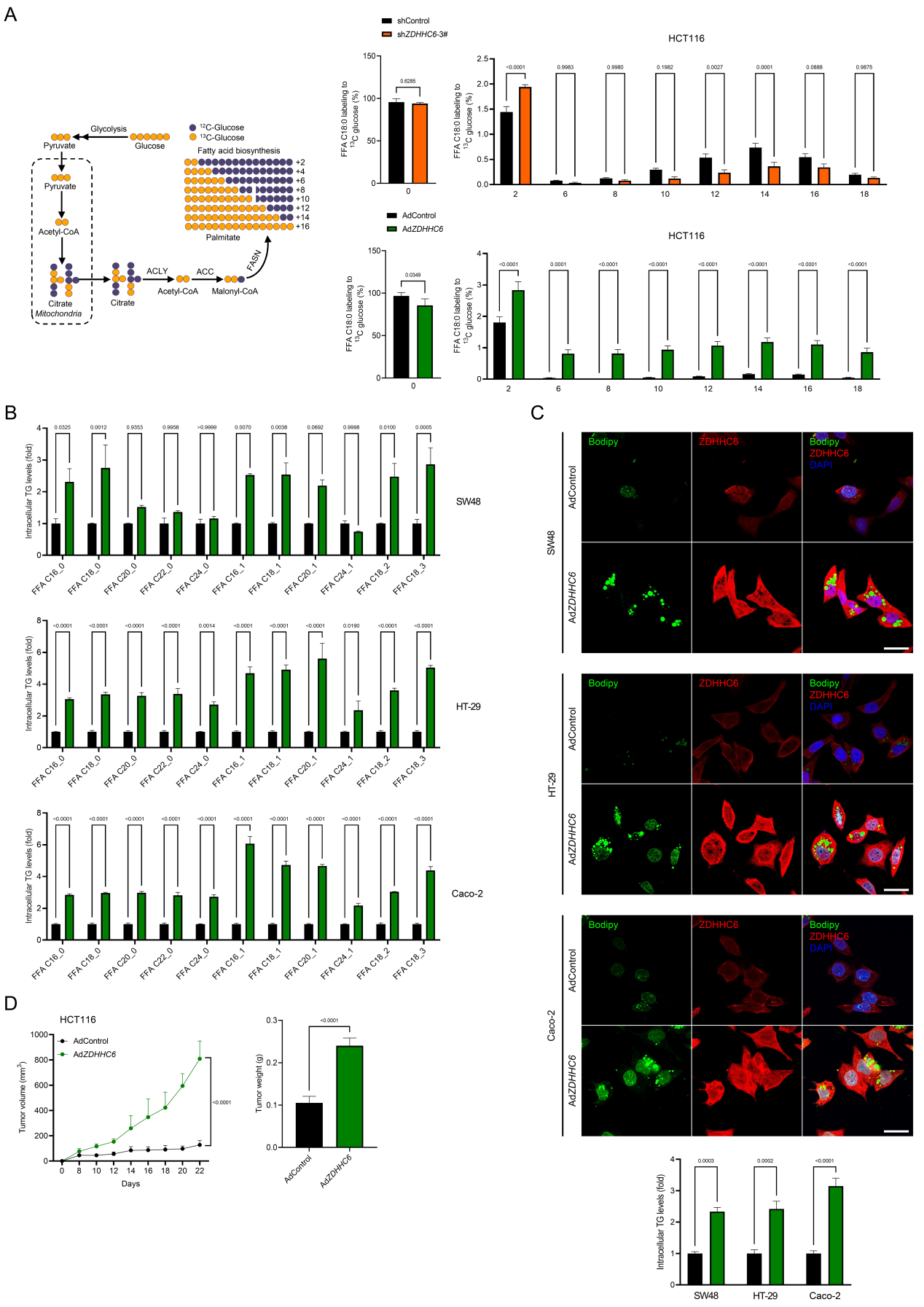


**Supplementary figure 4. ZDHHC6 promotes fatty acids biosynthesis and CRC carcinogenesis.**

(**A**) Diagram illustrating the production of fatty acids from ^13^C-glucose. The distribution of different isotopic forms of FFA C18:0 following the introduction of labeled glucose [U-^13^C] in HCT116 cells transfected with shZDHHC6 or Ad*ZDHHC6* for overexpression. The medium was switched to RPMI 1640 with 2 g/L glucose labeled with [U-^13^C] when the cell density reached around 80%. After 24 hours, the cell culture plates were rinsed with PBS, snap-frozen in liquid nitrogen, and analyzed using LC-MS. There are 5 individuals in each group.
(**B**) The levels of various FFAs were examined in SW48, HT-29, and Caco-2 cells using LC-MS-based untargeted metabolomic analysis. The results were normalized based on the overall peak area. There are 5 individuals in each group.
(**C**) Representative immunofluorescence images of SW48, HT-29, and Caco-2 cells overexpressing ZDHHC6, showing ZDHHC6 expression levels (red), lipid accumulation (Bodipy staining), and intracellular triglyceride (TG) levels. There are 5 individuals in each group.
(**D**) Nude mice were injected with HCT116 cells that overexpressed ZDHHC6 into their right flanks. Tumor volumes were measured every other day. On the 22nd day after dissection, growth curves and weight measurements were documented for each group consisting of 4 subjects. Scale bars, 1 cm.

Data are expressed as mean ± SEM. The relevant experiments presented in this part were performed independently at least three times. *P* <0.05 indicates statistical significance.
