# Supplementary material for: Palmitoyltransferase ZDHHC6 promotes colon tumorigenesis by targeting PPARγ-driven lipid biosynthesis via regulating lipidome metabolic reprogramming
Source: J Exp Clin Cancer Res. 2024 Aug 16;43:227. doi: 10.1186/s13046-024-03154-0 (PMC11328492; doi:10.1186/s13046-024-03154-0)
Supplement: Supplementary file 5 — Supplementary Material 5 [file 13046_2024_3154_MOESM5_ESM.docx]

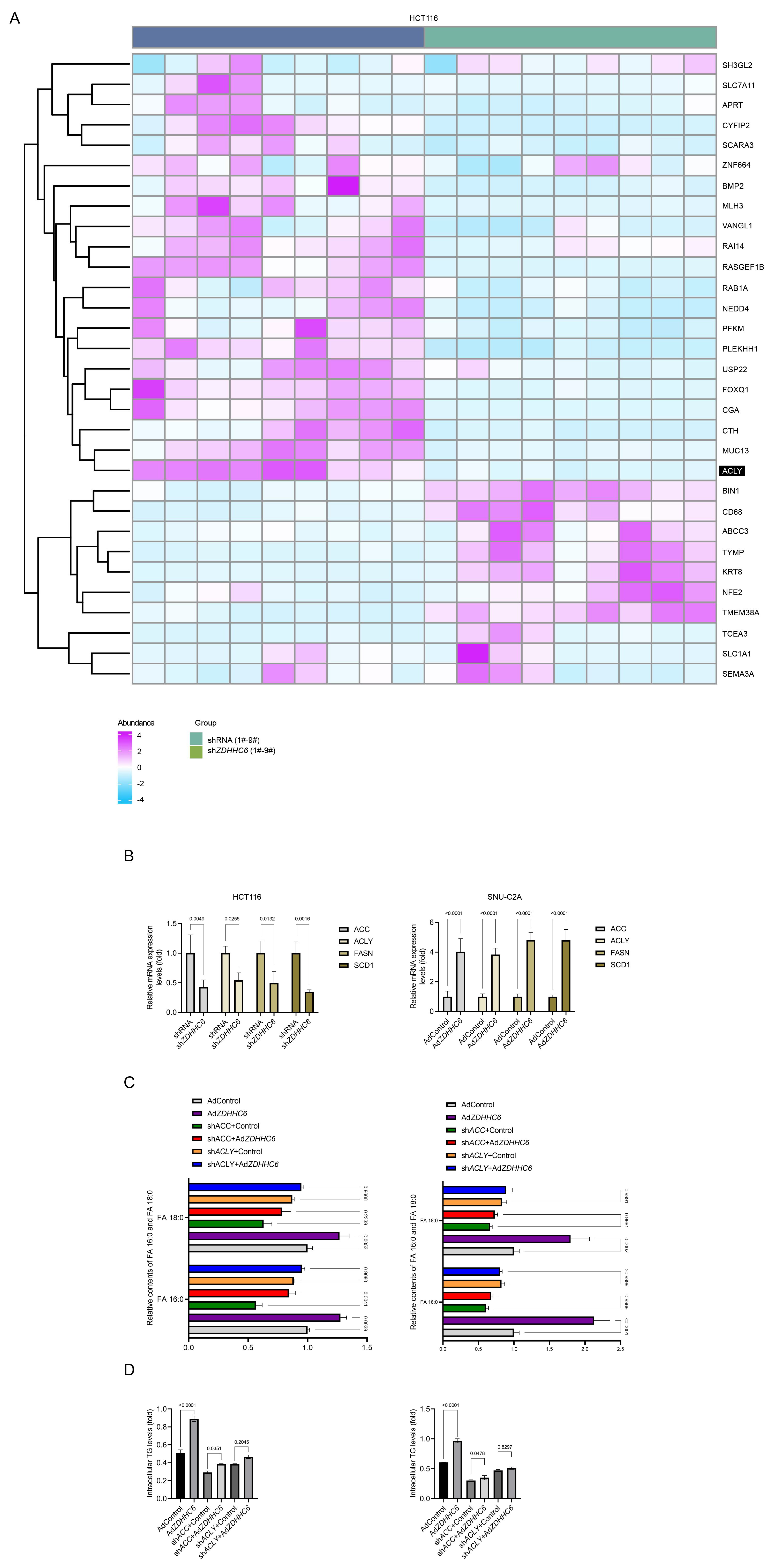


**Supplementary figure 5. ZDHHC6 increases ACC and ACLY expression.**

(**A**) A heatmap showing the 30 most downregulated and upregulated genes in HCT116 cells that were exposed to ZDHHC6 shRNA. The colors red, pink, and light blue indicate the Log2 fold change (Fc) representing either an increase or decrease in mRNA expression relative to the shRNA control group.
(**B**) Quantitative PCR was performed to analyze the expression of ACC, ACLY, FASN, and SCD in HCT116 cells with ZDHHC6 knockdown and SNU-C2A cells overexpressing ZDHHC6. There are 5 individuals in each group.
(**C**) The proportions of FFA C16:0 and FFA C18:0 were examined in HCT116 and SNU-C2A cells transfected with ZDHHC6 alone or in conjunction with ACC or ACLY shRNA. Cells were examined using liquid chromatography-mass spectrometry after adhering for 24 hours. There are 5 individuals in each group.
(**D**) The relative concentration of triglycerides was measured in the cell lines mentioned in (C). Cells were examined 24 hours after adhering. There are 5 individuals in each group.

Data are expressed as mean ± SEM. The relevant experiments presented in this part were performed independently at least three times. *P* <0.05 indicates statistical significance.
