# Supplementary material for: Palmitoyltransferase ZDHHC6 promotes colon tumorigenesis by targeting PPARγ-driven lipid biosynthesis via regulating lipidome metabolic reprogramming
Source: J Exp Clin Cancer Res. 2024 Aug 16;43:227. doi: 10.1186/s13046-024-03154-0 (PMC11328492; doi:10.1186/s13046-024-03154-0)
Supplement: Supplementary file 6 — Supplementary Material 6 [file 13046_2024_3154_MOESM6_ESM.docx]

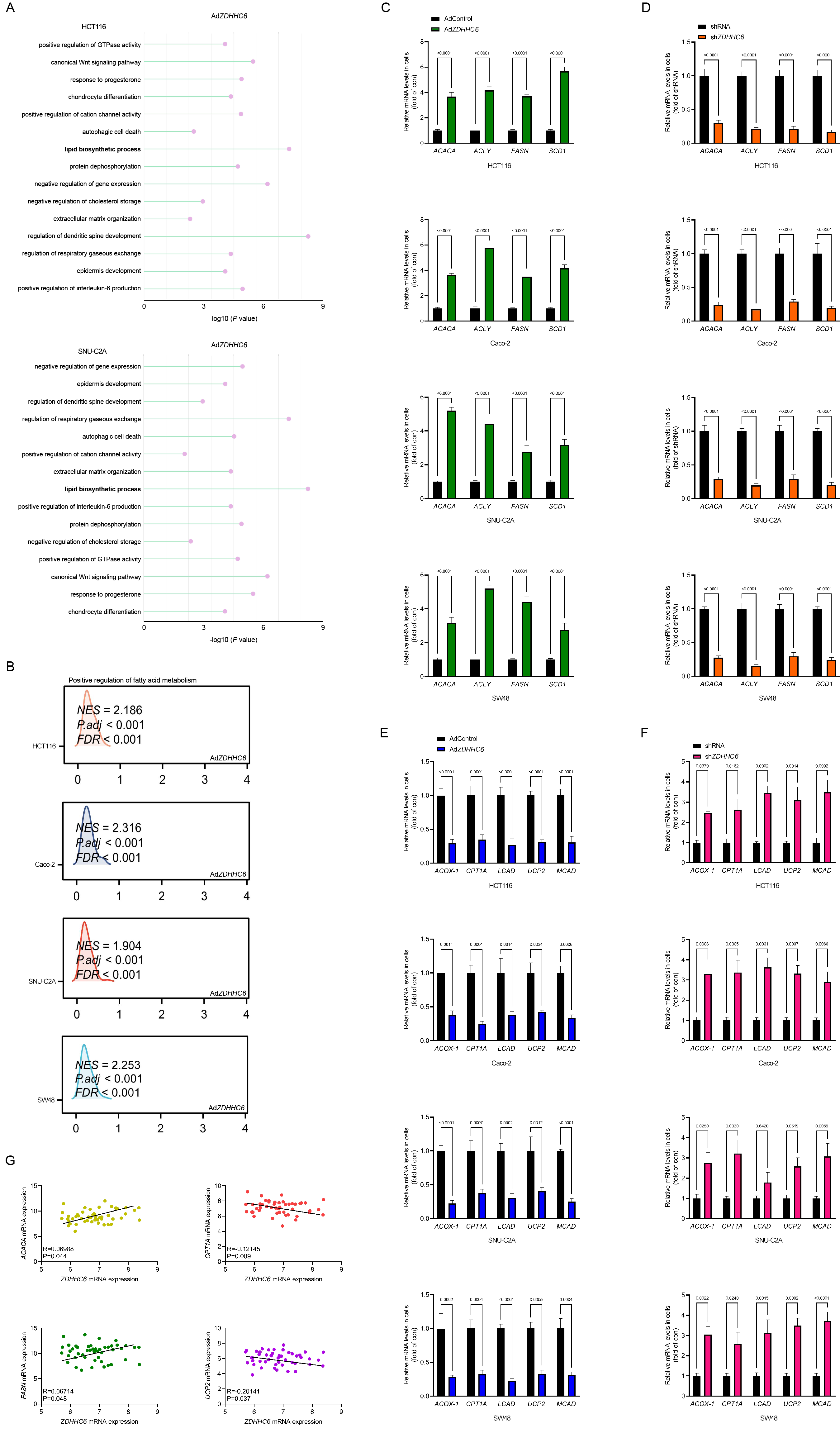


**Supplementary figure 6. ZDHHC6 upregulates ACC and ACLY expression.**

(**A**) Biological process analysis was conducted on genes showing substantial differences (Log2 Fc＞1.2) in the transcriptome data of HCT116 or SNU-C2A cells transduced with Ad*ZDHHC6*. The website link is https://david.ncifcrf.gov/.
(**B**) Performing gene set enrichment analysis on the expression profiles of HCT116, Caco-2, SNU-C2A, and SW48 cells transduced with Ad*ZDHHC6* to identify enriched positive fatty acid metabolism gene sets.
(**C, D**) Quantitative PCR was used to analyze the mRNA expression of ACACA, ACLY, FASN, and SCD1 in HCT116, Caco-2, SNU-C2A, and SW48 cells that were transduced with Ad*ZDHHC6* or shZDHHC6. Each group consisted of 5 samples.
(**E, F**) Quantitative PCR was used to analyze the mRNA expression of ACOX-1, CPT1A, LCAD, UCP2, and MCAD in HCT116, Caco-2, SNU-C2A, and SW48 cells that were transduced with Ad*ZDHHC6* or shZDHHC6. Each group consisted of 5 samples.
(**G**) Correlation analysis was conducted between ZDHHC6 and ACACA, CPT1A, FASN, and UCP2 using the TCGA CRC database. R denotes the Pearson correlation coefficient.

Data are expressed as mean ± SEM. The relevant experiments presented in this part were performed independently at least three times. *P* <0.05 indicates statistical significance.
