# Supplementary material for: Palmitoyltransferase ZDHHC6 promotes colon tumorigenesis by targeting PPARγ-driven lipid biosynthesis via regulating lipidome metabolic reprogramming
Source: J Exp Clin Cancer Res. 2024 Aug 16;43:227. doi: 10.1186/s13046-024-03154-0 (PMC11328492; doi:10.1186/s13046-024-03154-0)
Supplement: Supplementary file 8 — Supplementary Material 8 [file 13046_2024_3154_MOESM8_ESM.docx]

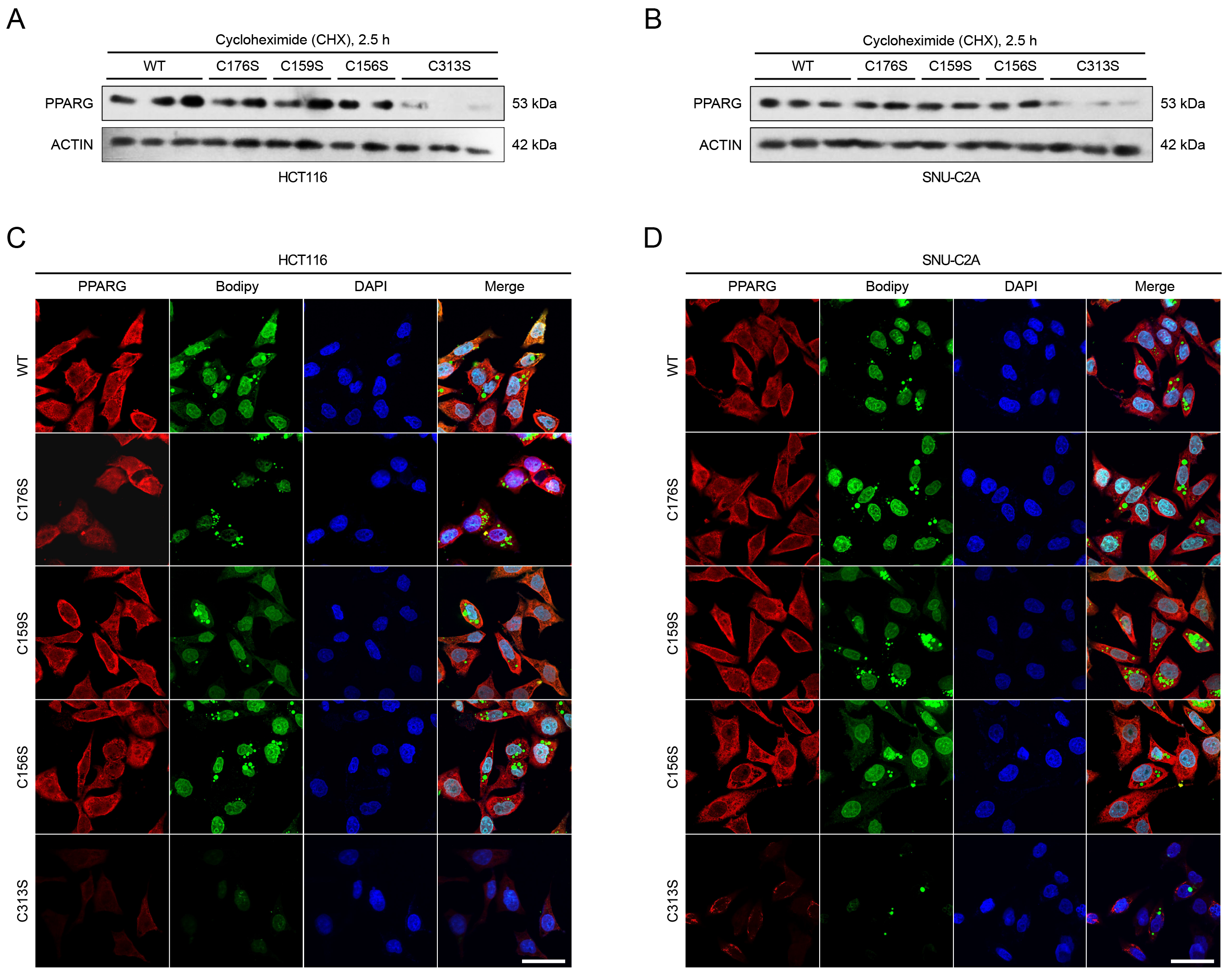


**Supplementary figure 7. Identification of cysteine 313 as the palmitoylation site on** **PPARγ.**

(**A**) The degradation of PPARγ WT, PPARγ C176S mutant, PPARγ C159S mutant, PPARγ C156S mutant, and PPARγ C313S mutant was seen in HCT116 and SNU-C2A cells following a 2.5-hour treatment with CHX. *n* = 3 per group.
(**B**) HCT116 and SNU-C2A cells transfected with specified mutant vectors were analyzed using immunofluorescence to identify PPARγ protein levels and lipid buildup. Tissue slices were stained with PPARγ (red) and lipid (Bodipy, green) using immunofluorescence. *n* = 10 per group. Scale bars, 20 μm.

Data are expressed as mean ± SEM. The relevant experiments presented in this part were performed independently at least three times. *P* <0.05 indicates statistical significance.
