# Supplementary material for: Palmitoyltransferase ZDHHC6 promotes colon tumorigenesis by targeting PPARγ-driven lipid biosynthesis via regulating lipidome metabolic reprogramming
Source: J Exp Clin Cancer Res. 2024 Aug 16;43:227. doi: 10.1186/s13046-024-03154-0 (PMC11328492; doi:10.1186/s13046-024-03154-0)
Supplement: Supplementary file 9 — Supplementary Material 9 [file 13046_2024_3154_MOESM9_ESM.docx]

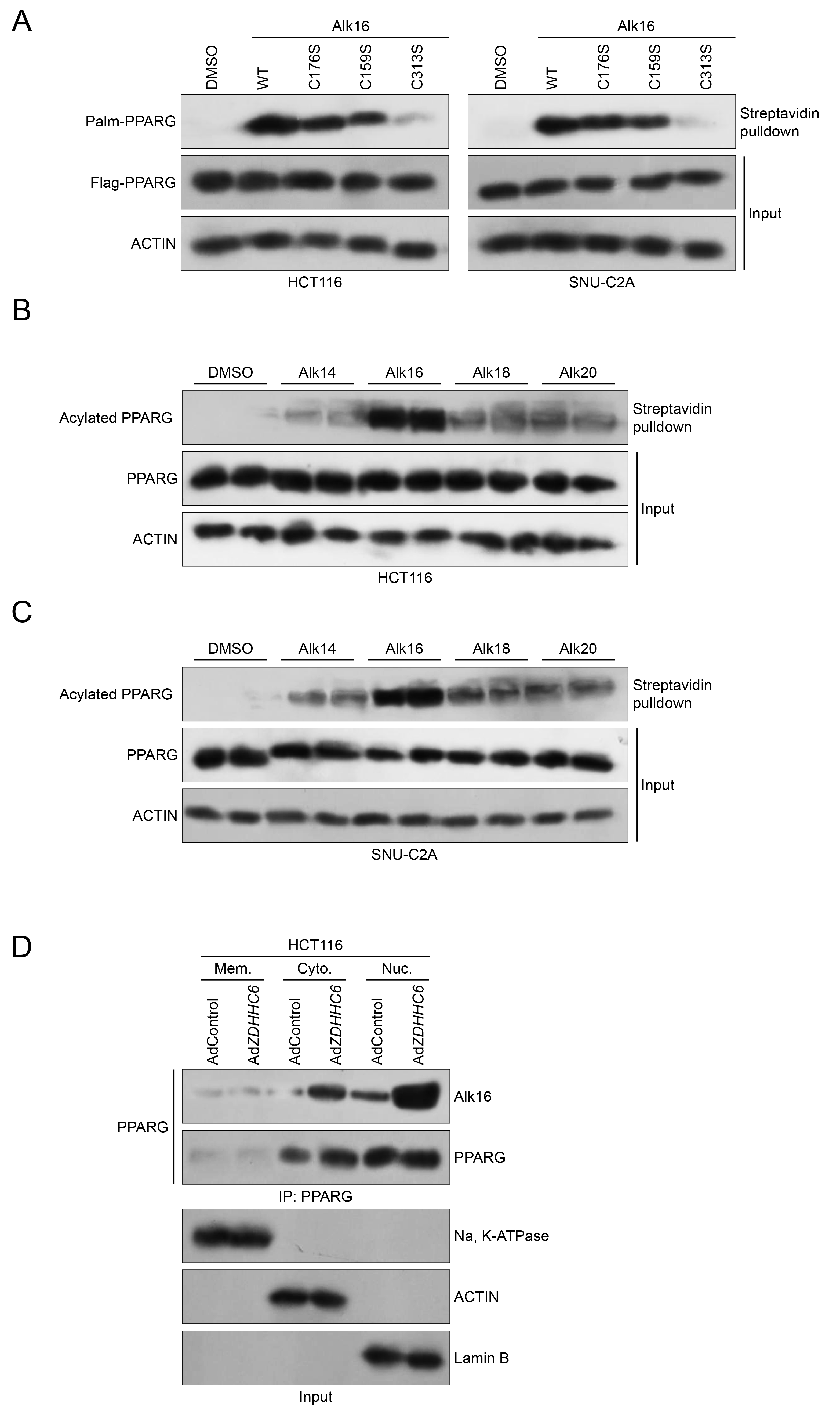


**Supplementary figure 8.** **PPARγ is palmitoylated at** **evolutionarily conserved cysteine residues in CRC cells.**

(**A**) Analyzed the amount of palmitoylation in Flag-labeled PPARγ WT, PPARγ C176S, PPARγ C159S, and PPARγ C313S mutants in HCT116 and SNU-C2A cells by labeling with Alk16 and using CLICK reaction-associated streptavidin pulldown. The lysates were analyzed using immunoblotting with Flag and ACTIN antibodies. There are 3 individuals in each group.
(**B**) Detection of PPARγ palmitoylation in HCT116 cells employing various alkyl-labeled fatty acylation, such as Alk14, Alk16, Alk18, and Alk20. Acylated PPARγ were identified by streptavidin bead pulldown, followed by immunoblotting with PPARγ and ACTIN antibodies. There are 6 participants in each category.
(**C**) The identical technique to (B) was utilized to identify acylated PPARγ in SNU-C2A cells. The lysates were analyzed using western blotting using PPARγ and ACTIN antibodies. There are 6 participants in each category.
(**D**) The wild-type HCT116 cells were transfected with PPARγ-Flag and then labeled with Alk16. Subcellular fractions were collected and PPARγ protein levels were adjusted to ensure equivalent amounts of PPARγ in the specified cell component for analysis. Immunoblotting analysis detected amounts of palmitoylated PPARγ in the cell membrane, cytoplasm, and nucleus.
