# Supplementary material for: Palmitoyltransferase ZDHHC6 promotes colon tumorigenesis by targeting PPARγ-driven lipid biosynthesis via regulating lipidome metabolic reprogramming
Source: J Exp Clin Cancer Res. 2024 Aug 16;43:227. doi: 10.1186/s13046-024-03154-0 (PMC11328492; doi:10.1186/s13046-024-03154-0)
Supplement: Supplementary file 10 — Supplementary Material 10 [file 13046_2024_3154_MOESM10_ESM.docx]

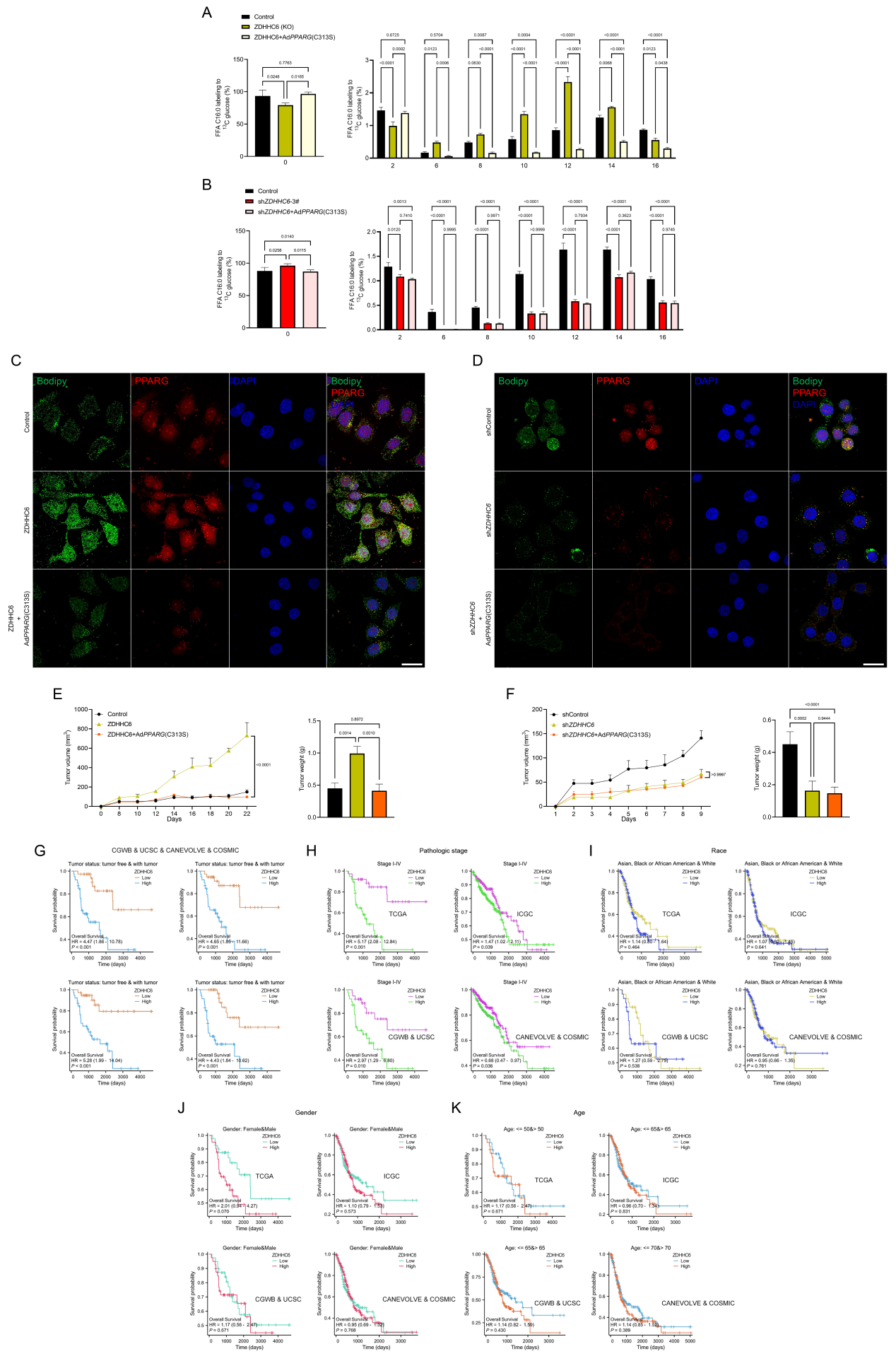


**Supplementary figure 9.** **ZDHHC6-mediated fatty acid biosynthesis promotes CRC carcinogenesis by upregulating PPARγ.**

(**A, B**) The distribution of several isotopomers of FFA C16:0 following labeling with [U-^13^C] glucose in HCT116-derived stable cells with different genetic modifications. The medium was switched to RPMI 1640 with 2 g/L glucose labeled with [U-^13^C] when the cell density reached around 80%. After 24 hours, the cell culture plates were rinsed with PBS, snap-frozen in liquid nitrogen, and analyzed using LC-MS. There are 5 individuals in each group.
(**C, D**) Immunofluorescence images show the relative TG content and abundance of PPARγ expression in the cell lines specified in (A) and (B). Each group consists of 5 members. The scale bars are 20 μm.
(**E**) In null mice, HCT116-related stable cells (Control, ZDHHC6 knockout, ZDHHC6+AdPPARγ C313S) were injected into the right flanks. Tumor volumes were measured every other day. Weight and tumor growth curves were assessed 22 days post-dissection. Each group has *n* = 5.

(**F**) In null mice, HCT116-related stable cells (Control, shZDHHC6, and shZDHHC6+AdPPARγ C313S) were implanted into the right flanks. Tumor volumes were measured every other day. Weight and tumor growth curves were assessed 22 days after dissection. Each group has *n* = 5.

(**G**) Kaplan-Meier survival analysis curves were generated to assess the prognostic significance of ZDHHC6 in four colorectal cancer databases: CGWB, UCSC, CANEVOLVE, and COSMIC.
(**H-K**) Kaplan-Meier curves of survival analysis of prognostic value of ZDHHC6 in stage I-IV (H), race (I), gender (J), and age (K) of CRC in different CRC databases including TCGA, ICGC, CGWB, UCSC, CANEVOLVE, and COSMIC.
Data are expressed as mean ± SEM. The relevant experiments presented in this part were performed independently at least three times. *P* <0.05 indicates statistical significance.
