# Supplementary material for: Palmitoyltransferase ZDHHC6 promotes colon tumorigenesis by targeting PPARγ-driven lipid biosynthesis via regulating lipidome metabolic reprogramming
Source: J Exp Clin Cancer Res. 2024 Aug 16;43:227. doi: 10.1186/s13046-024-03154-0 (PMC11328492; doi:10.1186/s13046-024-03154-0)
Supplement: Supplementary file 13 — Supplementary Material 13 [file 13046_2024_3154_MOESM13_ESM.docx]

**Palmitoyltransferase ZDHHC6 promotes colon tumorigenesis by targeting** **PPARγ-driven lipid biosynthesis via regulating lipidome metabolic reprogramming**

Junqi Shan^1^**^#^**, Xinyu Li^2^**^#^**, Runqi Sun^3^**^#^**, Yao Yao^1^, Yan Sun^4^, Qin Kuang^4^, Xianling Dai^4^, Yanlai Sun^1^*****

^1^ Department of Surgical Oncology, Shandong Cancer Hospital and Institute, Shandong First Medical University and Shandong Academy of Medical Sciences, Jinan 270000, Shandong, China

^2^ School of Clinical Medicine, Shandong Second Medical University, Jinan 270000, Shandong, China

^3^ School of Clinical Medicine, Jining Medical University, Jining 272000, Shandong, China

^4^ Key Laboratory of Biorheological Science and Technology (Chongqing University), Ministry of Education, College of Bioengineering, Chongqing University, Chongqing 400030, PR China

*****Corresponding author should be addressed to:

Prof. Yanlai Sun;

Department of Surgical Oncology, Shandong Cancer Hospital and Institute, Shandong First Medical University and Shandong Academy of Medical Sciences, Jinan 270000, Shandong, China

Email address: ylsun@sdfmu.edu.cn/ylsun_sdfmu@yeah.net

Tel/Fax:+86-053167626252

**^#^** These authors contributed equally to this work.

Junqi Shan, 15275043627@163.com;

Xinyu Li, 15088225385@163.com;

Runqi Sun, 3268837923@qq.com;

Yao Yao, 18363691964@163.com;

Yan Sun, 1125843281@163.com;

Qin Kuang, 1147744844@qq.com;

Xianling Dai, daixianling_cqu@qq.com;

Yanlai Sun, ylsun@sdfmu.edu.cn/ylsun_sdfmu@yeah.net

**Ethics approval and consent to participate**

The use of clinical samples was approved by the ethics committee of Shandong First Medical University & Shandong Academy of Medical Sciences and informed consent were obtained from all patients (2023LC21074). All animal experimental protocols were performed following the Ethical Animal Care and Use Committee of Shandong University (2021sa0541jk).

**Consent for publication**

All authors consent to the publication of this article.

**Availability of data and material**

All data and material during the current study are available from the corresponding author on reasonable request.

**Competing interests**

The authors declare no competing interests.

**Funding**

This work was supported by (1) the Start-up fund of Shandong Cancer Hospital (NO. 2020PYA05); (2) the National Science Foundation of Shandong Province (NO. ZR2020MH254) and (3) the Science and Technology Program of Jinan (NO. 202134062).

**Author contributions**

Conceptualization & Methodology, Junqi Shan, Yanlai Sun;

Investigation, Junqi Shan, Xinyu Li, Runqi Sun, Yao Yao, Yan Sun, Qin Kuang, Xianling Dai, Yanlai Sun;

Data analysis, Junqi Shan, Xinyu Li, Runqi Sun, Yao Yao, Yanlai Sun;

Funding acquisition, Project administration & Supervision, Yanlai Sun;

Writing-original draft, Junqi Shan, Xinyu Li, Runqi Sun, Yanlai Sun;

Writing review & editing, Junqi Shan, Xinyu Li, Yanlai Sun.

All authors have read and approved the final manuscript.

**Abbreviations**

CRC: Colorectal cancer

ZDHHC6: Zinc finger DHHC-type palmitoyltransferase 6

ATCC: American Type Culture Collection

PPARγ: Peroxisome proliferator-activated receptor gamma

FBS: Fetal bovine serum

IHC: Immunohistochemistry

TCGA: The cancer genome database

1. BP: 2-bromopalmitate

HA: Hyaluronic acid

CM: Cytomembrane

ACLY: ATP citrate lyase

SCD: Stearoyl-CoA desaturase

FASN: Fatty acid synthase

ICGC: International cancer genome consortium

GEO: Gene expression omnibus

**Abstract**

**Background**

The failure of proper recognition of the intricate nature of pathophysiology in colorectal cancer (CRC) has a substantial effect on the progress of developing novel medications and targeted therapy approaches. Imbalances in the processes of lipid oxidation and biosynthesis of fatty acids are significant risk factors for the development of CRC. Therapeutic intervention that specifically targets the peroxisome proliferator-activated receptor gamma (PPARγ) and its downstream response element, in response to lipid metabolism, has been found to promote the growth of tumors and has shown significant clinical advantages in cancer patients.

**Methods**

Clinical CRC samples and extensive in vitro and in vivo experiments were carried out to determine the role of ZDHHC6 and its downstream targets via a series of biochemical assays, molecular analysis approaches and lipid metabolomics assay, etc.

**Results**

To study the effect of ZDHHC6 on the progression of CRC and identify whether ZDHHC6 is a palmitoyltransferase that regulates fatty acid synthesis, which directly palmitoylates and stabilizes PPARγ, and this stabilization in turn activates the ACLY transcription-related metabolic pathway. In this study, we demonstrate that PPARγ undergoes palmitoylation in its DNA binding domain (DBD) section. This lipid-related modification enhances the stability of PPARγ protein by preventing its destabilization. As a result, palmitoylated PPARγ inhibits its degradation induced by the lysosome and facilitates its translocation into the nucleus. In addition, we have identified zinc finger-aspartate-histidine-cysteine 6 (ZDHHC6) as a crucial controller of fatty acid biosynthesis. ZDHHC6 directly interacts with and adds palmitoyl groups to stabilize PPARγ at the Cys-313 site within the DBD domain of PPARγ. Consequently, this palmitoylation leads to an increase in the expression of ATP citrate lyase (ACLY). Furthermore, our findings reveals that ZDHHC6 actively stimulates the production of fatty acids and plays a role in the development of colorectal cancer. However, we have observed a significant reduction in the cancer-causing effects when the expression of ZDHHC6 is inhibited in *in vivo* trials. Significantly, in CRC, there is a strong positive correlation between the high expression of ZDHHC6 and the expression of PPARγ. Moreover, this high expression of ZDHHC6 is connected with the severity of CRC and is indicative of a poor prognosis.

**Conclusions**

We have discovered a mechanism in which lipid biosynthesis is controlled by ZDHHC6 and includes the signaling of PPARγ-ACLY in the advancement of CRC. This finding provides a justification for targeting lipid synthesis by blocking ZDHHC6 as a potential therapeutic approach.

**Key words:** Colorectal cancer (CRC); ZDHHC6; PPARγ; Palmitoylation; Lysosomal degradation

**Introduction**

Colorectal cancer ranks as the third most prevalent form of cancer in the population and is well recognized as a significant global public health concern. Colorectal cancer has the potential to metastasize from the colon's surface to the colon tissue and other organs such as the liver, prostate gland, seminal vesicle gland, and uterus if not detected early ^1, 2^. While much research has been conducted on the molecular process behind the development of intestinal cancer, additional investigation is required to identify new and efficient therapeutic approaches. Recent epidemiological studies have discovered a connection between metabolic irregularities and numerous colon disorders, such as ulcerative colitis (UC), Crohn's disease (CD), and colorectal cancer (CRC) ^3, 4^. The colon, being a crucial organ for metabolizing the three macronutrients, has been identified as playing a significant role in these diseases. It is important to mention that the worldwide occurrence of CRC is experiencing a significant rise, primarily attributed to the widespread problem of being overweight and the consequent emergence and advancement of obesity-related generalized metabolic syndrome ^5, 6^. The lipid-rich state has a crucial role in promoting chronic inflammatory bowel disease (IBD) and colorectal cancer (CRC) driven by metabolic syndrome ^7, 8^. Recent reports have extensively characterized the genetic changes in colorectal cancer (CRC), including mutations in TP53, KRAS, and EGFR ^9, 10^. CRC is a very diverse malignancy with a low response to treatments and a high fatality rate. The etiology of colorectal cancer (CRC) and its related tumor formation is not yet fully comprehended, however disruption of genetic, immunological, and metabolic homeostasis is regarded to be crucial factors.

Metabolic reprogramming is primarily caused by the dysregulation of metabolic enzyme expression or activity. Palmitoylation is an essential posttranslational modification that controls the breakdown, location, and activation of metabolic enzymes in cells ^11, 12^. It involves attaching fatty acids to substrate proteins. The disruption of palmitoylation is intricately linked to cellular lipid metabolism and facilitates the development and advancement of several types of malignancies, including colorectal cancer (CRC) ^13^. Recently, there has been extensive research on a class of acyltransferases called zinc finger-aspartate-histidine-cysteine (DHHC)-CRD-type palmitoyl acyltransferases (ZDHHCs) due to their probable involvement in stabilizing oncoproteins ^14^. These enzymes have been investigated in several types of cancer. *S*-palmitoylation is a posttranslational modification that involves the attachment of C16 fatty acid palmitate (PA) to cysteine (Cys) residues of specific proteins ^14, 15^. This process regulates the functionality of these proteins in different pathological and physiological situations. Significantly, palmitoylation has been found to regulate the specific localization and movement of proteins. This biological process relies on the catalytic activity of a sequence of palmitoyltransferases. ZDHHC2 and ZDHHC3, which are clearly described enzymes, have significant involvement in the process of lipogenesis ^16, 17^. They directly palmitoylate and stabilize important lipogenesis components such as stearoyl-CoA desaturase (SCD), fatty acid synthase (FASN), and nuclear factor erythroid-2-related factor 2 (Nrf2) during carcinogenesis. ZDHHC6, a significant palmitoyltransferase, has been identified as a cancer signature gene and has high expression in various types of cancer ^18-20^. ZDHHC6 primarily functions as an oncoprotein by increasing AEG-1 palmitoylation, which in turn activates or stabilizes transcription factors involved in cancer progression ^21^. In addition, ZDHHC6 has a role in promoting cell proliferation by controlling the flow of calcium ions through selenoprotein K during the development of tumor growth ^22, 23^. Nevertheless, the role of ZDHHC6 in lipid metabolism is largely unknown.

Peroxisome proliferator-activated receptor gamma (PPARγ) plays a crucial role in controlling lipid metabolism and energy balance, which are important biological processes involved in the advancement of colorectal cancer (CRC) ^24, 25^. PPARγ has influence on diverse metabolic pathways and is present in colorectal epithelial cells, where its activation can stimulate cell differentiation and enhance apoptosis, therefore regulating tumor formation ^26^. Moreover, PPARγ has been recognized as a crucial agent in the process of adipocyte differentiation, lipid storage, and glucose metabolism, all of which play a significant role in creating the tumor microenvironment and facilitating the survival of cancer cells ^27, 28^. The abnormal lipid metabolism in cancer cells is currently acknowledged as a distinguishing feature of cancer and has a substantial impact on facilitating rapid cell growth and spread to other parts of the body ^29^. CRC cells frequently undergo significant modifications in both fatty acid synthesis and degradation, which play a role in membrane biosynthesis, energy storage, and signal transmission. These changes ultimately support the formation and progression of cancer. PPARγ agonists have been shown to inhibit the development of colon cancer, highlighting the receptor's potential as a target for therapeutic intervention in the treatment of colorectal cancer (CRC) ^30, 31^. Changes in PPARγ expression and activity can influence the development of colorectal cancer (CRC) by changing the way lipids are processed, causing inflammation, and impacting insulin sensitivity. These factors are crucial for cancer cells to adapt and survive in the intestinal environment. Nevertheless, the precise involvement of PPARγ in cancer biology is not well understood. Recent research have shown that the process of phosphorylation of AKT stimulates the production of PPARγ, which in turn enhances the synthesis of lipids and the development of tumors in colorectal cancer (CRC) ^32-34^. Our work revealed a correlation between the overexpression of ZDHHC6 and a substantial increase in lipid biosynthesis in CRC. We demonstrated that ZDHHC6 promotes the synthesis of fresh fatty acids and the formation of tumors by palmitoylating and stabilizing PPARγ in colon cancer. The results of our study offer a potential approach to specifically inhibit the production of fatty acids, which could have therapeutic advantages for individuals with elevated ZDHHC6 levels in colorectal cancer.

**Materials and Methods**

**Research consent and ethical statement**

The experimental procedures involving animals in this study were conducted in accordance with the guidelines outlined in the Guide for the Care and Use of Laboratory Animals (1996, in Chinese). Additionally, these procedures were approved by the Institutional Animal Use and Care Committee at Shandong Cancer Hospital and Institute, Shandong First Medical University, and Shandong Academy of Medical Sciences in Jinan, China. The study recruited patients with colorectal cancer (CRC) phenotypes from the Department of Gastrointestinal Surgery and Clinical Trial Research Center at Shandong Cancer Hospital and Institute, Shandong First Medical University, and Shandong Academy of Medical Sciences (Jinan, China). The diagnosis of colorectal cancer (CRC) was established after a comprehensive evaluation that included radiologic, clinical, and endoscopic examinations, as well as careful analysis of histology data. The individuals included in this study had been diagnosed with Crohn's disease (CD), ulcerative colitis (UC), or colorectal cancer (CRC). The determination of ulcerative colitis (UC) or Crohn's disease (CD) was achieved through the utilization of histological data in conjunction with radiologic, clinical, and endoscopic assessments. The specific attributes are outlined in Supplemental table 1 and 2. Each patient donor granted informed consent, and the research was approved by the Institutional Research Ethics Committee of Shandong First Medical University and Shandong Academy of Medical Sciences (Jinan, China).

**Identification of Candidates**

We screened candidate genes using the following procedures in order to find genes involved in the progression and metabolism of colorectal cancer (CRC): (1) Data Acquisition: The Cancer Genome Atlas (TCGA), International Cancer Genome Consortium (ICGC), and Gene Expression Omnibus (GEO) databases provided gene expression data and pertinent clinical information on CRC patients (GEO: GSE254054, GSE231943, GSE252858, GSE234804, GSE236678, GSE231436, GSE197088, and GSE239549). (2) Finding consistently differentially expressed genes (DEGs): In each data set, the function *t*-test in R was used to see if there was a significant difference in the level of gene expression between CRC tissues and adjacent or normal tissues. Benjamini-Hochberg correction was then applied. For the ensuing analysis, the lists of upregulated or downregulated DEGs in each of the ten datasets were utilized. (3) Candidate gene survival study in colorectal cancer: Clinical status and follow-up duration were taken from the clinical data of CRC patients. After excluding patients for whom there was no overall survival statistics or information, a survival analysis was carried out using the R packages "survival" and "survminer." (4) Gene set variation analysis (GSVA): The sample-wise biological processes of gene ontology (GO) activity variation were estimated using the GSVA R package. Single-sample GSVA scores were computed using a gene expression matrix, with each GO gene set including a minimum of ten genes. The GSVA scores of biological processes linked to cancer and the fold change in the expression levels of overlapping genes were analyzed using the Pearson correlation coefficient. (5) GSEA, or gene set enrichment analysis: In each dataset, CRC samples were compared to adjacent normal tissues using the GSEA software. The Molecular Signatures Database provided the gene set 'c5.all.v6.2.symbols.gmt', which was utilized in this investigation. Following the use of a 0.05 P value criterion and a 0.25 FDR threshold, the genes found were considered significant. Additionally, we ran GSEA on CRC tissues with high and low ZDHHC6 expression levels from the TCGA and ICGC databases.

**Cell Culture**

The human colorectal cancer cell lines SNU-C2A (Cat#: CCL-250.1), SW48 (Cat#: CCL-231), HT-29 (Cat#: HTB-38), LS1034 (Cat#: CRL-2158), HCT116 (Cat#: CCL-247EMT), Caco-2 (Cat#: HTB-37), and the FHC cells (Cat#: CRL-1831) derived from the human colon were acquired from the American Type Culture Collection (ATCC). The mouse-associated CRC cell lines, namely CT26 (Cat#: CRL-2638), MC38 (Cat#: CVCL_B288), CMT93 (Cat#: CVCL_1986), MC26 (Cat#: CVCL_0240), and the normal mouse colon cell line MODE-K (Cat#: CVCL_B4FG), were acquired from Cellosaurus. The entire cell line was discovered and confirmed to be devoid of mycoplasma. CRC cell lines were cultured in RPMI1640 medium (Cat#: LM87077C, LMAI Bio, China) supplemented with 10% premium quality fetal bovine serum (FBS) (Cat#: abs972, Absin, China) and 1×penicillin-streptomycin solution (Cat#: U31-301C, YOBIBIO, China). The cells were cultured in culture plates at 37°C in a humidified atmosphere with 5% CO2. The FHC or MODE-K cells were cultured in RPMI-1640 media supplemented with 10% fetal bovine serum (FBS), as specified in the product and instruction manuals. Following the transfection process, specific cells were placed in 6-well, 12-well, or 24-well plates with 60% fusion for 24 hours prior to cell transfection. Subsequently, the cells were transfected using Convoy^TM^ Transfection Reagent (Cat#: No. 11103, ACTGene Inc.) as instructed in the product description. Concisely, the transfection mixture was formulated by combining 2 μg of vector, 5 μl of Lipo3000 transfection reagent, and 120 μl of Gibco Opti-MEM™.

**Antibodies, Chemicals and Reagents**

The corresponding primary antibodies used in this study were purchased from Abcam Inc®. The antibodies were specific to the following target proteins: ACTIN (Catalog number: ab179467, diluted at a ratio of 1/2500), PPAR gamma (Catalog number: ab272718, diluted at a ratio of 1/1000), PPAR delta (Catalog number: ab23673, diluted at a ratio of 1/1000), PPAR alpha (Catalog number: ab61182, diluted at a ratio of 1/1000), SREBP1 (Catalog number: ab28481, diluted at a ratio of 1/1000), Myc (Catalog number: ab32, diluted at a ratio of 1/1000), RAB11B (Catalog number: ab228954, diluted at a ratio of 1/150-1/1000), LAMP1 (Catalog number: ab24170, diluted at a ratio of 1/150-1/1000), Lamin B1 (Catalog number: ab229025, diluted at a ratio of 1/1000). The antibodies against ZDHHC6 (Catalog number: ABIN7162558, dilution ratio of 1/200–1/1000) and ZDHHC6 (Catalog number: ABIN6989238, dilution ratio of 1/200–1/1000) were acquired from antibodies-online in Limerick, PA, US. The antibodies against HA (Catalog number: 3724, diluted at a ratio of 1/250–1/1000) and Flag (Catalog number: 14793, diluted at a ratio of 1/250–1/1000) were acquired from Cell Signaling Technology™ (CST). The supplementary anti-Na, K-ATPase antibody (Catalog number: H00000483-B01P) and PPARG antibody (Catalog number: NBP2-22106) were acquired from Novus Biologicals, LLC at a dilution ratio of 1/250-1/1000.

The protein concentration of the samples was determined using the BCA Protein Assay Kit (Catalog number: YSD-500T, Yoche-Biotech, China). The western blotting study employed HRP-tagged secondary antibodies (Catalog number: bs-0297M-HRP, Bioss, China) at a dilution ratio of 1/15,000–1/20,000 for visualization purposes. The MG-132 (Catalog number: T2154) and cycloheximide (CHX) (Catalog number: T29590) were acquired from TargetMol Chemicals Inc., located in Shanghai, China. The compounds Biotin picolyl azide (Catalog number: 900912), alkynyl myristic acid (Alk14) (Catalog number: 1164), alkynyl palmitic acid (Alk16) (Catalog number: 1165), and alkynyl stearic acid (Alk18) (Catalog number: 1166) were acquired from Click Chemistry Tools. We synthesized and manufactured the alkynyl arachidic acid (Alk20) in our laboratory, achieving a purity level of above 98%. The compounds 2-bromopalmitate (2-BP) (Catalog number: 21604) and BSA (Catalog number: V900933) were acquired from Merck KGaA. The compound known as palmostatin M (Palm M) (Catalog number: 565407) was acquired from MedKoo Biosciences, Inc. The TaqMan® Universal PCR Master Mix (Catalog Number: P/N4304437) and PowerUp™ SYBR™ Green (Catalog Number: A25742) were acquired from Applied Biosystems.

The pan palmitoylation-specific antibody was acquired as a generous gift from the research laboratory of Dr. Xu at the Chongqing University of Education ^35^. In summary, to create an antibody that specifically targets pan palmitoylation, a brief peptide containing just two Cys C-C (pal) bonds (98.5% purity) was produced. One of the cysteine residues in the peptide was palmitoylated. This peptide was then utilized as the hapten, following established techniques and procedures. Subsequently, the acquired peptide C-C (pal) was linked with keyhole limpet hemocyanin (KLH) to serve as an extra antigen for the immunization of New Zealand white rabbits. The anti-serum was collected following the administration of three doses of passive immunization. Prior to utilization, employ ammonium sulfate precipitation for the purification of this anti-serum. In order to enhance the verification of this antibody, we have employed multiple supplementary validation methodologies. Essentially, the hydroxylamine buffer liquid can generate free sulfhydryl groups through the thioester linkages formed between the protein and palmityls. The TS-6B resin was employed to isolate the palmitoylated valosin-bearing protein that had been treated in a hydroxylamine buffer. Subsequently, an immunoblotting experiment was conducted using an anti-valosin-bearing protein antibody. Additionally, the acquired antibody was subsequently employed to examine the palmitoylation of the short c-terminal domain phosphatase 1, as detailed in the prior investigation. In this study, the pre-existing antibody was used to examine the palmitoylated proteins obtained from cells treated with various drugs, including Palm B, Palm M, or 2-BP. The aforementioned techniques collectively enhanced the sensitivity and specificity of the antibody in detecting the protein palmitoylation process.

**Knockout Cell and Knockdown Lines Preparation**

The pre-existing system for generating knockout cell lines with targeted gene deficiencies, which is relevant to the ongoing research, was established as previously described. Essentially, a CRC cell line was created by using the CRISPR-Cas9 method to delete the ZDHHC6 or PPARG genes. The pre-designed short guide RNA (sgRNA) targeting the human *ZDHHC6* or *PPARG* gene was generated and inserted into pLentiCRISPRV2-GFP vectors (#98290, Testobio Co., Ltd., China) to produce the CRISPR-Cas9 sgRNA-packaging lentivirus. The ready-made CRISPR/Cas9 KO products for human ZDHHC6 plasmid (#sc-418298) and PPARγ plasmid (#sc-400030) were acquired from Santa Cruz Biotechnology, Inc. The sgRNA oligonucleotides were inserted into pLentiCRISPRV2 vectors that had been cleaved by the BsmBI restriction enzyme. The clones carrying gene deletions were extracted and identified using western blotting.

**Plasmids and Vectors Preparation**

The segments of DNA encoding the sgRNAs were inserted into PX330 (#PVT6301, Nova Lifetech Inc.) in order to introduce the N-terminal S-protein-Flag-Streptavidin binding peptide (SFB) tag at the endogenic *ZDHHC6* gene location. The donor vector for *ZDHHC6* knock-in was created using Gibson assembly of 50 homologous arms, Puro-P2A-SFB, and 30 homologous arms into the pUC19 plasmid (#N3041L, New England Biolabs). The *ZDHHC6* open reading frames of human origin were produced using PCR amplification using complementary DNA derived from HCT116 cells. In addition, full-length *Homo sapiens* *ZDHHC6* cDNA plasmids were created using PCR-based cDNA amplification. These plasmids were then inserted into pcDNA3.4™ TOPO™ plasmids (#A14697, Thermo Fisher Scientific Inc.) that were tagged with either 3×HA or 3×Flag for use in other *in vitro* investigations. Expression vectors containing shortened fragments of the *PPARG* gene, such as Myc-PPARG WT, Myc-PPARG-AF1, Myc-PPARG-DBD, and Myc-PPARG-hinge-LBD, were generated as planned. In addition, to further investigate the functional impacts of ZDHHC6 in laboratory conditions, targeted protein expression vectors were created by loading adenoviruses with the use of a pre-made adenovirus packaging kit (Haixing Biosciences, Suzhou, China). The complete *Homo sapiens ZDHHC6* cDNA plasmids and their corresponding ready-made shRNA targeting human *ZDHHC6* (sh*ZDHHC6*), shRNA targeting human *PPARG* (sh*PPARG*), human *PPARG* sequences with the C313S mutant, and *Homo sapiens* full-length *PPARG* sequences were individually packaged into adenovirus using a commercially available adenovirus packaging kit (Haixing Biosciences, Suzhou, China). The adenovirus particles were purified and quantified to a titer of 6.0×10^10^ PFU using the Adenovirus Purification Mini Kit (#V1160-01, Shanghai Juncheng Biotechnology, China) following the instructions provided in the operation handbook.

**Histological Analysis**

The histopathologic assay was conducted by fixing the cells or tumor tissue with a 4% formaldehyde solution called Image-iT™ (#R37814, Thermo Fisher Scientific Inc.), and thereafter cutting it into transverse sections. The cell slices were subjected to treatment with the specified primary antibodies at a temperature of 4 °C for a duration of 24 hours. The Leica DM IL LED microscope was used to capture histological pictures for examining specimen sections, while a confocal laser microscopy system (Olympus, Japan) was used for investigating immunofluorescence sections after incubating them with the appropriate secondary antibodies. The tumor specimens were immersed in a 4% formaldehyde solution for 24 hours to preserve them, and subsequently embedded in an OCT (optimal cutting temperature) compound. The embedded tissues were sliced into sections of 20 micrometers in thickness. Xylene was employed for the purpose of eliminating the paraffin from the tissue sections during the process of IHC staining, followed by subsequent rehydration. Subsequently, they were immersed in a solution containing 5% normal target serum and 3% BSA (Beyotime) for a duration of 1.5 hours. The specified primary antibodies were introduced and left to incubate overnight at a temperature of 4°C. Following the washing step, a 1:300 dilution of HRP-conjugated secondary antibody (Abcam) was applied and incubated for 1 hour. The DAB substrate was introduced to analyze the signals. Photographs were taken with a light microscope.

**Immunoprecipitation and *S*-Protein pull down assay**

Immunoprecipitation and SFB pull-down experiment was performed as described previously ^42^. Briefly, cells were lysed in E1A lysis buffer (250 mM NaCl, 50 mM HEPES [pH 7.5], 0.1% NP-40, 5 mM EDTA, protease inhibitor cocktail [Sigma]). The antibodie to ZDHHC6 was used for immunoprecipitation. HCT116 cells were transfected with SFB-tagged protein and lysed in NETN buffer (200 mM Tris-HCl [pH 8.0], 100 mM NaCl, 0.05% NP-40, 1 mM EDTA, protease inhibitor cocktail [Sigma]) for 20 min at 4 °C. Crude lysates were subjected to centrifugation at 14,000 × g for 15 min at 4 °C. Supernatants were incubated with S-Protein Agarose for 4 h (Millipore, USA). The agaroses were washed three times with NETN buffer. Proteins were eluted by boiling in 1× SDS loading buffer and subjected to SDS-PAGE for immunoblotting.

**GST pull-down assay**

GST pull-down assay was used to detect the direct interaction between PPARγ and ZDHHC6. Briefly, GST-tagged PPARγ (GST-PPARγ) and 6×His-tagged ZDHHC6 (His-ZDHHC6) or 3×Flag-tagged ZDHHC6 (Flag-ZDHHC6) proteins were expressed in BL21 (DE3) Escherichia coli via transforming pGEX-4T-1-GST-PPARγ and pET24a-His-ZDHHC6 or pET24a-Flag-ZDHHC6 plasmids, respectively. Then, the E. coli were collected, sonicated, and purified with cOmplete His-Tag Purification Resin (Roche) or Purification of Flag kit (Sigma-Aldrich) to obtain purified ZDHHC6-tagged protein. GST-PPARγ protein was expressed and immobilized with BeyoGold™ GST-tag Purification Resin (Beyotime) following the manufacturer’s instructions. The beads-PPARγ complexes were washed with GST pull-down binding buffer (50 mM Tris-HCl, 200 mM NaCl, 1 mM EDTA, 1% NP-40, 1 mM DTT, 10 mM MyCl2, pH 8.0) and incubated with purified ZDHHC6-tagged protein at 4 °C for 4 h on a rotating windmill. Finally, the beads were washed and analyzed by immunoblotting assay.

**Quantitative PCR (****qPCR) Assay**

The cells or tissue RNA were obtained and isolated using TRNzol universal reagent (#DP405-02, TIANGEN Biotech Co., Ltd., Beijing) following the instructions provided in the operating handbook. The pre-prepared RNA samples were stored in a refrigerator at a temperature of -80 °C for a maximum of 10 days (#TSX40086V, Thermo Fisher Scientific Inc.). The optimal RNA purity was assessed based on the observation of an absorption ratio (values >2.00) at 260/280 nm. Subsequently, 1.5 μg of pure RNA was subjected to reverse transcription using the PrimeScript™ RT-PCR Kit (#RR014A, Takara) and the Fast PCR Master Mix (#HY-K0532, MedChemExpress, Beijing). The reverse transcription process was conducted at a temperature of 42°C for a duration of 1 hour, and subsequently, the enzyme was deactivated at a temperature of 70°C for a period of 10 minutes. The PCR procedure was conducted utilizing SYBR Green qPCR Master Mix (#HY-K0501, MedChemExpress, Beijing) and SYBR qPCR Master Mix (Universal) (#22204, ToloBio, China) on QuantStudio 7 Pro equipment (Applied Biosystems). The pre-designed, precise primer sequences were acquired from OriGene Technologies, Inc.

**Western Blotting Assay**

In order to conduct the western blotting assay, the materials were prepared by homogenizing them with a commercially available RIPA lysis buffer solution (#BL504A, Biosharp, Shanghai, China). Subsequently, the ultimate homogenized extraction was condensed using centrifugation at a temperature of 4 °C and a speed of 13000 rpm for a duration of 30 minutes. The protein samples obtained were standardized using the BCA Protein Assay Kit (Catalog number: YSD-500T, Yoche-Biotech, China), using lipid-free BSA as the control. The cell lysis samples were analyzed using a 12% ShineGelTM Plus Tris-Glycine PAGE kit (#SP0511, Shinegene, China) and transferred onto a 0.45 μM hydrophobic polyvinylidene fluoride transfer membrane (#IPFL00010, Sigma-Aldrich). Immunoblotting was performed using the specified primary antibodies. Afterwards, the PVDF membranes were subjected to treatment with Western Blocker™ buffer in a working solution of 1×TBS, which included 0.1% Tween-20 (#T104863, Aladdin, China), for a duration of 1 hour. Subsequently, the membranes were incubated with the designated primary antibodies at a temperature of 4°C for a period of 24 hours. The PVDF membranes used in Western blotting were subjected to exposure using the ECL Plus kit test (#E266188, Aladdin, China) and subsequently exposed to medical X-ray film (Blue Ocean Imaging Systems, China). The protein contents were quantified as gray value levels using Image J, version 1.8.0, and then normalized to ACTIN as a fold change.

**Labelling, Click Chemistry, and Palmitoylation Identification**

The Click-iT™ Palmitic Acid (PA), Click-iT™ Cell Reaction Buffer Kit (#C10269), Click-iT™ Protein Reaction Buffer Kit (#C10276), and Azide Kit (#C10265) were acquired from Invitrogen for the purpose of conducting click chemistry and identifying palmitoylated PPARG, following the instructions provided with the products. Following the specified transfection of PPARG WT and PPARG C313S mutants, the cell media was treated with 100 μm Click-iT PA, azide, and kept in a 5% CO_2_, 37 °C environment for 5 hours. After a treatment duration of 5 hours, the cells were collected and subsequently rinsed four times with pre-cooled D-PBS (#abs9340, Absin, China). This was followed by a combination with lysis buffer containing 1×protease and a general phosphatase inhibitor cocktail (#abs9162, Absin, China). The lysates were thereafter kept at a temperature of 4 °C for a duration of 40 minutes, after which they were transferred to a centrifugal tube with a volume of 2 ml. The ultimate cell lysates were condensed using centrifugation at a temperature of 4 °C and a speed of 13500 revolutions per minute for a duration of 10 minutes. The protein concentration of the collected supernatant was verified using the EZQ™ Protein Quantitation Kit (#R33200, Thermo Fisher Scientific) following the instructions provided in the product manuals. The extracted proteins were incubated with biotin-alkyne using the Click-iT™ Protein Reaction Buffer Kit, following the protocols outlined in the production instruction specification. Subsequently, the biotin-alkyne-palmitic acid and azide-protein complexes were subjected to streptavidin-mediated pulldown experiments using streptavidin magnetic beads (#HY-K0208, MedChemExpress, China). The resulting samples were then analyzed by western blotting using the PPARG antibody.

Furthermore, to enhance the identification of the palmitoylation of PPARG, an additional complementary approach was employed in this section. The cells were treated and lysed using a buffer containing 1% Triton X-100, 150 mM NaCl, 0.2% SDS, 50 mM TEA-HCl at pH 7.4. The buffer also contained 1×protease and phosphatase inhibitor cocktail. Additionally, a click reaction with biotin azide was performed. The cell proteins were extracted using 10 volumes of 100% methyl alcohol at a temperature of -80 °C for a duration of 1.5 hours. Subsequently, the proteins were collected again by centrifugation at a speed of 13500 revolutions per minute for a period of 15 minutes. The precipitates were resuspended in 100 ml of a 1×suspension buffer and subsequently diluted to a 12-fold immunoprecipitation solution. The immunoprecipitation solution consisted of 0.5% Nonidet P 40, 55 mM Tris-HCl, 160 mM NaCl, 6.5 mM EDTA, and had a pH of 7.5. The cell proteins that were marked with labels were concentrated using streptavidin agarose at a temperature of 30 °C, with a gentle swirling motion for a duration of 1.5 to 2.0 hours. The protein-bound streptavidin agarose beads underwent four washes using an immunoprecipitation solution. Subsequently, the protein-bound beads were released using an elution buffer consisting of 95% formamide and 10 mM EDTA with a pH of 8.2, heated to 95 °C for a duration of 5 minutes. The samples were then analyzed using immunoblotting detection.

**Fractionation Assay**

The separation of components was achieved using the Nuclear/Cytosol Fractionation Kit (#K266-100, BioVision, US) or Nuclear/Cytosol Extraction Kit (#ab289882, Abcam). The nuclear component was disrupted using a homogenization solution consisting of 5% SDS, 1×protease inhibitor cocktail, 2.5 mM PMSF, 160 mM NaCl, 60 mM triethanolamine, 1600 units/ml benzonase nuclease, pH 7.5. After removing the solution, a final homogenization of 6.5 mM was achieved by adding EDTA. Prior to the subsequent fractionation assay, the cytoplasmic component was diluted using a homogenization solution that contained 6 mM EDTA.

**Cell Proliferation Detection**

The CCK-8 assay was used to measure the proliferation of cancer cells. The cultured cells were placed in a 96-well plate and incubated for different lengths of time, with a density of 2.5×10^4^ cells per well. After each treatment, the liquid above the culture was removed, and 15 μl of Cell Counting Kit-8 (CCK-8) from ApexBio Technology (#K1018) was added to each well. After being kept at a temperature of 37°C for 2 hours, the fluorescence intensity at a wavelength of 450 nm was quantified using a microplate reader. Colony formation was utilized as a technique to examine cell proliferation. To summarize, the designated cancer cells were inserted in a 24-well plate, with each well containing 200 cells. Following a period of 2 weeks, colonies with a cell count exceeding 50 were visually observed and measured using crystal violet staining.

**Transwell Assay**

Cell migration and invasion were quantified using Transwell assay. The Corning® Transwell® (#CLS3422, Corning, USA) was cultured with Matrigel (#356234, BD, USA). The tumor cells that were identified were cultivated again in a medium without serum (#12-725F, LONZA, USA) and then placed in the upper chamber with a density of 6.0×10^4^ cells per well. Subsequently, the medium containing 10% fetal bovine serum (FBS) was introduced into the bottom chamber, and then administered for a duration of 48 hours. Subsequently, the cells in the top chamber were entirely eliminated by means of a cotton swab. The cells that moved into the lower chamber were washed, treated with a 4% formaldehyde solution to preserve them, and then colored with a 0.65% crystal violet stain. Finally, the cells were visualized using a microscope. The migration calculation experiment was conducted using identical techniques, with the exception that Matrigel was omitted during the pre-coating of the upper chamber.

***In vivo* Xenograft Models Construction**

Pathogen-free male BALB/c nude mice (5 weeks old, 20-22 g, Beijing Vital River Laboratory Animal Technology Co., Ltd., Beijing, China) underwent a subcutaneous procedure in the flank region, where 1.6×10^6^ HCT116 or SNU-C2A cells were injected in a suspension of 80 µl PBS. The mice were split into treatment groups at random once the xenograft tumors reached the expected volume. Tumor dimensions were measured at regular intervals of 2-3 days using a vernier caliper. The tumor's volume was determined by applying the formula 1/2×D (major axis)×d2 (minor axis). Mice were euthanized without causing pain after the tumor size reached 2 cm^3^ or when ulceration was detected. All animal experimental protocols were performed following the Ethical Animal Care and Use Committee of Shandong University (2021sa0541jk).

**Triglyceride Detection and Lipid Accumulation In Vitro Assay**

The measurement of TG was conducted using the Triglyceride Quantification Kit (#K622-100, BioVison, US). A total of 6×10^5^ cells were gathered and subsequently combined with 1 ml of extraction reagent. The samples were subjected to ultrasonic treatment for a duration of 1 minute, followed by centrifugation at a speed of 9000×g at a temperature of 4°C for a duration of 10 minutes. The liquid portion was collected for analysis in accordance with the guidelines provided by the manufacturer. The Green Fluorometric Lipid Droplet Assay Kit (BODIPY-493/503) was employed to detect lipid deposition in CRC cells. This kit allowed visualization of the production of lipid droplets in the specified in vitro tests.

**RNA Sequencing**

The TRNzol universal reagent was used to extract RNA from HCT116 cells, following the manufacturer's procedure. The quantification of RNA samples was performed using the BioPhotometer Plus (Eppendorf), while the assessment of RNA integrity was conducted using the Agilent 2100 bioanalyzer system. The Collibri Stranded RNA Library Prep Kit for Illumina, provided by Thermo Fisher Scientific, was utilized for RNA sequencing library preparation, following the instructions provided by the vendor. The sequencing process was performed on the Illumina HiSeq instrument utilizing a 2 × 150 Paired End configuration, with 35 million reads obtained per sample by GENEWIZ, LLC. The construction of sequencing libraries was performed using the Small RNA-Seq Library Prep Kit (#052.08, Lexogen GmbH, Vienna, Austria) with total RNA as the starting material. In summary, the messenger RNA (mRNA) was extracted from the whole RNA using Sera-Mag SpeedBead particles and subsequently subjected to chemical fragmentation. The RNA fragments were subjected to reverse transcription, where they were converted into complementary DNA (cDNA) using random primers that had a tagging sequence attached to their 3′ ends. The cDNA libraries were subsequently amplified using the KAPA high-fidelity DNA polymerase. The validation of the libraries' quality was conducted using the Agilent 2100 bioanalyzer. Following that, a high-throughput sequencing procedure was carried out utilizing an Illumina NovaSeq 6000. Following the mapping of sequences using the STAR or HISAT2 software against Homo sapiens or Mus musculus, a biological pathway analysis was conducted using Cluster-Profiler. The RNA sequencing and library creation were carried out by the technical personnel at Nanjing Biomed Sciences Research Bio-Lab.

**Metabolomics and Lipidomics Assay**

Approximately 30 milligrams of tissues were combined with 550 microliters of a 75% methanol buffer to extract metabolites. The liquid portion was collected and subjected to lyophilization. The freeze-dried powder was dissolved in 60 μL of 80% methanol. Following centrifugation, the liquid remaining after sedimentation, known as the supernatant, can be used for analysis with the TSQ Fortis Plus (#TSQ03-10003, Thermo Fisher Scientific)-Orbitrap Astral High Resolution MS (Thermo Fisher Scientific). The process of extracting metabolites from cells involved the following steps: Firstly, cells collected in a 10-cm dish were washed with pre-cooled PBS and immediately frozen using liquid nitrogen. The cells were subsequently disrupted using 1.5 mL of 80% methanol solution containing internal standards, and then removed from the dish by scraping. The liquid portion obtained after spinning the mixture at high speed and removing the solid particles was preserved. This liquid was then subjected to a process of removing moisture by freezing and subsequent drying. The resulting dried material was used for the examination of small molecules involved in metabolism using a technique called liquid chromatography-mass spectrometry (LC-MS). The process of extracting lipids from cells was conducted as follows: briefly, cells that were gathered in a 10-cm dish were washed with PBS and immediately frozen using liquid nitrogen. The cells were subsequently disrupted using 1.5 mL of methanol containing internal standards, combined with 1.5 mL of chloroform, and vigorously mixed for 30 seconds using a vortex. Afterward, 500 µL of water were added and vortexed for an additional 30 seconds. The layer that repels water was gathered and subjected to freeze-drying. The dehydrated powder was reconstituted in 40 μl of an organic solvent mixture (Chloroform: Methanol, 2:1) using a vortex for 30 seconds, and then combined with 70 μl of another organic solvent mixture (Acetonitrile: Isopropanol: Water, 13:6:1). Following centrifugation, the liquid portion can be specifically utilized for TSQ Fortis Plus-Orbitrap Astral MS analysis.

**Glucose [U-^13^C] Tracer Assay**

The medium was replaced with RPMI 1640 supplemented with glucose (2 g/L) labelled with [U-^13^C] when the cell density reached around 80%. After 24 hours, the cell culture plates were rinsed with PBS, rapidly frozen in liquid nitrogen, and kept at a temperature of -80 °C.

**Statistical analysis**

The data were expressed as the mean ± SEM (standard error of the mean) of three independent experiments conducted in triplicate. The software GraphPad Prism 8.0, developed by GraphPad Software in San Diego, USA, was utilized for the purposes of creating visual representations and doing statistical analysis. Unpaired Student's t-tests were employed to compare the two groups. A unidirectional analysis of variance (ANOVA) was conducted, followed by a Bonferroni post-hoc test to compare different groups. A *P* value less than 0.05 was deemed to be statistically significant. The scientists were unaware of the animal genotype and grouping information.

**Results**

**Identification of potential genes strongly associated with the course and prognosis of colorectal cancer (CRC)**

We examined the expression matrix of colorectal cancer and adjacent healthy tissues in public datasets that included high-coverage gene profiling data in the TCGA, ICGC, and NCBI Gene Expression Omnibus (GEO) databases (GEO: GSE254054, GSE231943, GSE252858, GSE234804, GSE236678, GSE231436, GSE197088, and GSE239549) in order to identify intriguing genes that regulate the progression of colorectal cancer (Figure 1A). The CRC tissues exhibited a substantial enrichment of events linked to tumors and processes related to metabolism (Figure 1B). Across the ten datasets we selected, we identified 41 differentially expressed genes (DEGs) that were conservatively upregulated and 101 DEGs that were downregulated (Figure 1C). Notably, based on clinical data from the TCGA and ICGC databases, 6 upregulated and 4 downregulated DEGs were identified because they showed a substantial correlation with survival rates (Figure 1D). Additionally connected to molecular processes influencing the development of colorectal cancer and metabolic reprogramming are these 6 upregulated and 4 downregulated DEGs (Figure 1E).

Of the top four elevated DEGs most closely connected with CRC occurrences, we concentrated on ZDHHC6, the sole gene having the most underappreciated biological significance in CRC (Figure 1E). Using the TCGA and ICGC datasets, we further confirmed that CRC had higher levels of ZDHHC6 expression than surrounding tissues (Figure 1F, G). The samples in the TCGA and ICGC databases were split into two groups based on the ZDHHC6 expression value: one group was ZDHHC6 high-expression, and the other ZDHHC6 low-expression. Crucially, compared to those with low ZDHHC6 expression levels, those with high ZDHHC6 expression levels had a noticeably worse survival prognosis (Figure 1H). There were significantly more biological processes associated with cancer growth and metabolic reprogramming in the group with high ZDHHC6 expression on a regular basis. This demonstrated that ZDHHC6 is potentially associated with biological processes of CRC (Figure 1I).

In addition, we examined the potential involvement of ZDHHC6 expression in colitis and the advancement of colorectal cancer (CRC) related with colitis, by analysing the intersection of ZDHHC6 in many datasets. Colon samples obtained from persons diagnosed with Crohn's disease (CD), ulcerative colitis (UC), and colorectal cancer (CRC), as well as from healthy donors (HC), exhibited markedly elevated levels of ZDHHC6 protein compared to the healthy individuals in the control group. This was particularly accurate for individuals with symptoms of ulcerative colitis (UC) and Crohn's disease (CD). Individuals exhibiting characteristics of colorectal cancer (CRC) demonstrated elevated levels of ZDHHC6 protein content in comparison to those in the Crohn's disease (CD), ulcerative colitis (UC), and healthy control (HC) groups (Supplementary figure 1A). The Pearson multiple correlation and multiple linear regression analyses demonstrated a positive correlation between ZDHHC6 protein levels and plasma concentrations of ESR, CRP, and ProCT. These laboratory markers are commonly used in the diagnosis of clinic colitis (Supplementary figure 1A, B). Significantly, we also verified a negative correlation between the levels of albumin (ALB) in the bloodstream, a potential indicator of inflammatory bowel disease (IBD), and the levels of ZDHHC6 in the colon. The results suggest a link between inflammatory bowel disease (IBD) and the concentrations of ZDHHC6 in the tissue of the colon (Supplementary figure 1A, B). A similar observation was documented in mice with colitis caused by dextran sulphate sodium (DSS), a frequently employed chemically-induced experimental model for colitis. Also, a notable elevation in ZDHHC6 protein levels as the disease advanced, as observed using the western blotting experiment (Supplementary figure 1C). Furthermore, considering the various physical characteristics and underlying factors that worsen both sudden and long-lasting inflammation of the colon, the objective of this study was to investigate whether changes in ZDHHC6 expression played a role in the advancement of colorectal cancer (CRC) and the development of chronic colitis caused by DSS. The control mice were given AOM+DSS treatment for a period of ten weeks to promote the onset of colitis-associated carcinogenesis (Supplementary figure 1D). Not surprisingly, there was a noticeable and significant increase in ZDHHC6 expression in colon samples from the mouse model with the CRC phenotype (Supplementary figure 1D). These findings indicated that abnormally upregulated ZDHHC6 levels are correlated with the severity of colitis and the development of CRC.

**Disturbed lipid metabolism in human CRC with upregulated** **ZDHHC6 levels**

In addition to the substantial rise in ZDHHC6 observed in the TCGA dataset (Figure 1G) and the ICGC database (Supplementary figure 2A), In order to confirm the increased expression of ZDHHC6 in colorectal cancer (CRC), we obtained 73 pairs of CRC samples and their matching adjacent samples. Our analysis revealed a significant increase in the mRNA expression levels of ZDHHC6 in CRC tissues compared to nearby normal tissues (Figure 2A). In addition, the analysis of proteins revealed that both human CRC tumour tissues and CRC-related cell lines (SNU-C2A, SW48, HT-29, LS1034, HCT116, and Caco-2) as well as mouse-associated colon cancer cell lines (CT26, MC38, CMT93, and MC26) exhibited elevated levels of ZDHHC6 expression. This finding was further validated through western blotting and immunofluorescence detection (Figure 2B-E, Supplementary figure 2B). Hence, we examined potential processes that could contribute to the overexpression of ZDHHC6 in colorectal cancer (CRC). Subsequently, we identified the changes in its expression profile when exposed to 2-bromopalmitate (2-BP), a broad inhibitor of protein palmitoylation. The ZDHHC6 protein expression in human CRC cell lines was significantly decreased in a dose-dependent manner when exposed to a concentration gradient of 2-BP, as shown in Figure 2F and G. In addition, the administration of 2-BP leads to the inhibition of ZDHHC6, which is directly associated with the decrease in Ki67-positive colon cancer cells. This correlation is supported by the results of an *in vitro* immunofluorescence experiment (Figure 2H).

Considering the significant impact of ZDHHC6 on the advancement of colorectal cancer (CRC) and its probable involvement in the metabolic processes of tumours, we further examined the influence of ZDHHC6 on the molecular metabolism of CRC. To characterise the disrupted metabolic processes in colorectal cancer (CRC), a high-throughput metabolomics analysis was conducted to identify the metabolites that were significantly altered in 10 sets of CRC and adjacent normal tissues (Figure 3A, B). In comparison to the adjacent normal tissues, there are 39 metabolites that exhibit significant changes in the cancerous tissues. The elevated metabolites consist primarily of lipids and lipid-like compounds, specifically fatty acids (FAs), phosphatidylcholine (PC), phosphatidylethanolamine (PE), lysophosphatidylethanolamine (LPE), sphingomyelin (SM), and lysophosphatidylcholine (LPC) (Figure 3C). An additional analysis was conducted to determine the enrichment of specific pathways utilising 39 metabolites that showed differential expression. The results indicated that the production of triacylglycerol, glycerol phosphate shuttle, and palmitoylated protein were enriched (Figure 3D, E). The findings validated that the lipidome composition exhibited a substantial increase in CRC tissues. However, the potential explanation for this abnormally vigorous lipid metabolism in CRC tissues remains uncertain. To investigate the potential connection between the abnormally increased lipidome in CRC and the expression of ZDHHCs, we analysed the expression of ZDHHCs in the aforementioned pairings of cancerous and neighbouring normal tissues. Our findings revealed that ZDHHC6 was the ZDHHC member with the highest level of expression, as shown in Figure 3F. The modified expression of ZDHHC6 was strongly associated with the synthesis of lipids and lipid-like metabolites in SNU-C2A and HCT116 cells when ZDHHC6 was either suppressed (sh*ZDHHC6*) or enhanced (Ad*ZDHHC6*) (Supplementary figure 2C, D). The Pearson multiple correlation and multiple linear regression analyses revealed a significant association between the levels of ZDHHC6 protein and various tumour markers, including CA125, CA50, CA724, CA199, CEA, CA242, CK-BB, and HCG (Figure 3G, H). These findings indicate a potential correlation between elevated levels of ZDHHC6 and an aberrant rise in lipid and lipid-like metabolites in CRC. However, the precise function of ZDHHC6 in lipid metabolism in CRC remains uncertain.

**ZDHHC6 enhances lipid deposition and carcinogenesis in CRC cells**

Consistent with our previous findings, we conducted additional experiments to specifically determine the function of ZDHHC6 in colorectal cancer (CRC). We investigated the impact of introducing Ad*ZDHHC6* or Adsh*ZDHHC6* into SNU-C2A and HCT116 cells on their rate of cell division (Supplementary figure 3A, B). Interestingly, SNU-C2A and HCT116 cells that were transfected with AdshZDHHC6 showed a significant decrease in cell viability and EdU staining intensity. Conversely, cells transfected with Ad*ZDHHC6* exhibited an increase in cell viability and EdU intensity. These findings suggest that alterations in ZDHHC6 expression in CRC cells may have an impact on proliferation in a laboratory setting (Supplementary figure 3C, D). Furthermore, our current study supports the notion that ZDHHC6 may play a role in the development of colorectal cancer (CRC) pathogenesis. This is consistent with our findings, as demonstrated by the transwell analysis, which showed a significant decrease in the invasion and migration of tumor cells when ZDHHC6 was silenced, compared to the control group (Supplementary figure 3E, F). Consistent with expectations, the levels of epithelial-mesenchymal transition (EMT)-related markers such as vimentin, N-cadherin, fibronectin, TGFβ1, and MMP13 were significantly reduced in SNU-C2A and HCT116 cells. Conversely, the expression of E-cadherin was greatly increased (Supplementary figure 3G, H). Furthermore, *in vivo* experiments demonstrated that inhibiting ZDHHC6 effectively decreased the rates of tumor growth and the weights of tumors in mice that had been implanted with the SNU-C2A tumor models (Supplementary figure 3I). The findings confirmed that the lack of ZDHHC6 played a role in inhibiting the growth of colon cancer cells and the process of EMT. Afterward, we discovered 36 metabolites that showed substantial changes in *ZDHHC6*-deletion CRC cells. Fatty acid metabolites exhibited a considerable drop in HCT116 cells lacking *ZDHHC6*. Furthermore, we conducted pathway enrichment analysis on the set of 36 metabolites and observed a significant association between ZDHHC6 and the pathways related to fatty acid production (Figure 4A-C). Glucose is a crucial source for the creation of fatty acids through *de novo* biosynthesis. Subsequently, we employed glucose [U-^13^C] to monitor the process of fatty acid production. Our findings revealed that the cells with *ZDHHC6* knockdown exhibited a substantial reduction in the labeling of palmitic acid and stearic acid from the glucose trackers (Figure 4D, Supplementary figure 4A). In contrast, the HCT116 cells that had an enhanced expression of ZDHHC6 showed a considerable increase in the labeling of palmitic acid and stearic acid from glucose (Figure 4E, Supplementary figure 4A). In addition, ZDHHC6 markedly enhanced the formation of triglycerides in HCT116 cells, as demonstrated by the bodipy staining experiment (Figure 4F). Additionally, we conducted analogous tests on SW48, HT-29, and Caco-2 cell lines. We noted a substantial increase in the expression of several fatty acids and triglycerides in the colorectal cancer cells that were overexpressing ZDHHC6 (Supplementary figure 4B, C). Collectively, these findings offer proof that ZDHHC6 is crucial in the buildup of lipid content and may stimulate the production of fatty acids from scratch in CRC cells.

Subsequently, we conducted experiments involving both loss-of-function and gain-of-function assays on CRC cell lines to validate the involvement of ZDHHC6 in carcinogenesis. To investigate the role of ZDHHC6 in colorectal cancer (CRC) cells in living organisms, we implanted *ZDHHC6*-deficient HCT116 cells and *ZDHHC6*-overexpressing HCT116 cells under nude mice. Tumor sizes and weights were significantly reduced in animals implanted with *ZDHHC6*-deficient HCT116 cells compared to those implanted with control cells (Figure 4G, H). In contrast, the upregulation of ZDHHC6 enhanced the tumorigenic capacity of HCT116 cells (Supplementary figure 4D). Conversely, the tumours originating from *ZDHHC6*-deleted HCT116 cells exhibited a notable reduction in several lipid metabolites, such as fatty acids, when compared to tumours formed from control cells (Figure 4I). In summary, our data validate that ZDHHC6 significantly contributes to lipid buildup and carcinogenesis in CRC.

**ZDHHC6 specifically targets and interacts with lipid metabolism key transcription factor of PPARγ**

To explore the impact of ZDHHC6 on lipid accumulation in colorectal cancer (CRC), we conducted high-throughput RNA sequencing analysis to examine the possible effects of ZDHHC6 on key enzymes involved in fatty acid synthesis in CRC cells. Out of the top 36 genes that showed significant differences in expression, previously identified genes involved in the production of free fatty acids (FFA) were shown to be responsive to the depletion of ZDHHC6 (Supplementary figure 5A). The overexpression of ZDHHC6 was found to be linked to the process of lipid biosynthesis, as proven by GO-biological process analysis (Supplementary figure 6A). Subsequent GSEA analysis revealed that the gene sets related to the production of fatty acids were considerably increased in HCT116, Caco-2, SNU-C2A, and SW48 cells that were transduced with ZDHHC6 overexpression, as supported by Supplementary figure 6B. The abnormal increase in fatty acid synthesis is strongly associated with the main metabolic enzymes ACLY, ACC, FASN, and SCD1. To validate the role of ZDHHC6 in regulating fatty acid metabolic genes, we conducted qPCR analysis on CRC cells with ZDHHC6 knockdown and cells with ZDHHC6 overexpression. The mRNA levels of ACC, ACLY, FASN, and SCD1 were reduced in the ZDHHC6-knockdown HCT116 and SNU-C2A cells, but increased in the ZDHHC6-overexpressing cells (Supplementary figure 5B). In addition, we conducted a thorough investigation to determine the potential involvement of ZDHHC6 in the regulation of fatty acid degradation. To achieve this, we analyzed the expression of enzymes related to fatty acid degradation (ACOX-1, CPT1A, LCAD, UCP2, and MCAD) in stable cells engineered with ZDHHC6. We employed qRT-PCR and observed that ZDHHC6 indeed influenced the expression of these enzymes in CRC cells (Supplementary figure 6C-G). To verify the role of ZDHHC6 in promoting lipidome accumulation via ACC and ACLY, we introduced ZDHHC6 into HCT116 and SNU-C2A cells that had been depleted of ACC or ACLY. Our findings indicate that the introduction of ZDHHC6 did not significantly enhance the buildup of intracellular fatty acids and triglycerides in the ACC or ACLY knockdown cells, as observed in cells transfected with ZDHHC6 alone (Supplementary figure 5C, D). Collectively, these findings suggest that ZDHHC6 enhances the accumulation of lipid molecules by upregulating the expression of ACC and ACLY in colorectal cancer.
 Subsequently, to determine the precise substrate targeted by ZDHHC6, which has the ability to control the production of ACC and ACLY, we employed a method of isolating the ZDHHC6-associated protein complex in HCT116 cells using tandem affinity purification, followed by analysis using mass spectrometry. Notably, PPARγ (PPARG) was ranked at the top of the list (Figure 5A). In addition, our research revealed that PPARγ is the sole counterpart among many transcription factors associated with lipid metabolism, such as PPARα, PPARδ, and SREBF1 (Figure 5B). Furthermore, endogenous PPARγ was found in endogenous ZDHHC6 immunoprecipitates from SNU-C2A, SW48, HT-29, LS1034, and Caco-2 cells (Figure 5C). Through in vitro pulldown tests using purified recombinant proteins, it was shown that ZDHHC6 directly interacts with PPARγ, as depicted in Figure 5D. Given significant potential correlation of ZDHHC6 with PPARγ, we next integrated the ZDHHC6-interacting proteins obtained from the IP-MS experiment and the upregulated proteins revealed by proteomics analysis, and we identified PPARγ as the protein that interacts with and is regulated by ZDHHC6 using human HCT116, Caco-2, SNU-C2A and HT-29 colon cancer cell lines (Figure 5E). Moreover, to further demonstrate the specific target of ZDHHC6 responsible for its specific function, we performed an additional immunoprecipitation-MS (IP-MS) analysis of Flag-ZDHHC6 and His-PPARγ-transfected HCT116, Caco-2, SNU-C2A, and HT-29, respectively, and identified 126 detectable potential ZDHHC6-interacting proteins. We then integrated the ZDHHC6-interacting proteins obtained from the IP-MS experiment and the upregulated proteins revealed by proteomics analysis, and we identified PPARγ as the key protein that interacts with and is regulated by ZDHHC6 (Figure 5E). On the other hand, Flag-ZDHHC6 and His-PPARγ were overexpressed in HCT116, Caco-2, SNU-C2A, and HT-29, respectively. Co-immunoprecipitation (Co-IP) experiments demonstrated that ZDHHC6 co-immunoprecipitated with PPARγ, and vice versa (Figure 5F). Glutathione *S*-transferase (GST) pull-down assay suggested that ZDHHC6 interacts directly with PPARγ in transfected Caco-2 and SNU-C2A cells, respectively (Figures 5G). This GST pull-down result is consistent with the direct protein interaction of ZDHHC6 and PPARγ in HCT116 cells (Figure 5D). The immunofluorescent staining test demonstrated a localization of ZDHHC6 (major in the cytoplasm) and PPARγ (major in the nucleus) mostly within the CRC cells (Figure 5H). According to reports, PPARγ is composed of three distinct structural domains: AF-1, DBD, and the hinge region. Pull-down experiments demonstrated a robust association between ZDHHC6 and the DBD fragment of PPARγ, but only a few interactions were observed with the hinge-region fragment (Figure 5I). In addition, an examination of the TCGA-CRC and ICGC-CRC databases revealed a substantial correlation between ZDHHC6 and the PPARγ pathway in CRC, as demonstrated in Figure 5J. In summary, our findings validate that ZDHHC6 has a unique interaction with PPARγ, a crucial transcription factor involved in lipid metabolism.

**ZDHHC6 palmitoylates and stabilizes** **PPARγ**

Given the strong association between ZDHHC6 and PPARγ, as well as the crucial role of ZDHHC6 as a palmitoyltransferase in various biological processes, we hypothesized that ZDHHC6 may regulate the palmitoylation of PPARγ in response to alterations in lipid metabolism that occur during the progression of CRC. Therefore, to determine the role of palmitoylated PPARγ in the advancement of CRC, we analyzed its expression patterns and changes in response to 2-bromopalmitate (2-BP), a broad inhibitor of protein palmitoylation. Upon incubation of HCT116 cells with 2-BP to decrease the levels of palmitoylated proteins, there was a significant decrease in the expression of PPARγ, both in terms of protein abundance and pan-palmitoylation contents (Figure 6A). In contrast, the expression levels of PPARγ in HCT116 cells were increased by the overexpression of palmitoylation by the use of palmostatin B (Palm B), palmostatin M (Palm M), or ABD957, which is an inhibitor of depalmitoylase enzymes. This increase in expression levels was confirmed through colocalization immunofluorescence analysis (Figure 6A). In order to assess the impact of 2-BP on the decrease of PPARγ expression, a click chemistry assay was utilized to visualize the palmitoylated PPARγ (Figure 6B). The presence of palmitoylation on PPARγ was verified using the streptavidin beads-mediated pulldown and western blotting analysis, as anticipated (Figure 6B).
 Furthermore, given the significant role of palmitoylation in the control of PPARγ stability, we were compelled to determine the precise position of palmitoylation on PPARγ. The function investigation involved determining the anticipated location of the palmitoylation site on PPARγ for *Homo sapiens* and *Mus musculus*. This was achieved through a combined analysis of the GPS-Palm software (MacOS_20200219) developed by The CUCKOO Workgroup (http://gpspalm.biocuckoo.cn/) and the MDD-Palm algorithm (http://csb.cse.yzu.edu.tw/MDDPalm/). Both methods fortuitously anticipated and furnished the foremost 4 palmitoylation sites for PPARγ, exhibiting distinct confidence intervals and quality ratings. It is worth mentioning that cysteine 313 (C313) in human PPARγ and mouse PPARγ were identified as the most probable and dependable location for protein palmitoylation modification (Figure 6C). Furthermore, the cysteine residue location exhibited significant conservation across many species taxa, as depicted in Figure 6C. The presence of PPARγ protein in HCT116 and SNU-C2A cells was confirmed by detecting the residual PPARγ WT, C176S, C159S, C156S, and C313S mutants following CHX injection. As predicted, only the C313S mutation showed a significant decrease in the amount of PPARγ, while the other variants did not show any significant changes (Supplementary figure 7A, B). The impact of a sequence of genetic alterations on the buildup of lipids in the specified transfected colorectal cancer (CRC) cells was additionally validated using immunofluorescence analysis (Supplementary figure 7C, D). Substitution of the C313 residue with serine completely inhibited the palmitoylation of PPARγ, as demonstrated by the Click-iT chemistry experiment (Figure 6D). The C313S mutant significantly decreased the expression of PPARγ protein profiles without affecting its mRNA levels (Figure 6D). The PPARγ with C313S mutant underwent degradation in parallel with CHX treatment, leading to a progressive reduction in the remaining PPARγ levels (Figure 6E), which was comparable to the impact of 2-BP administration. Considering the identification of C313 as the primary location for PPARγ palmitoylation, we are curious about the extent to which S-palmitoylation at C16 contributes to the chemical alteration of PPARγ. Hence, a sequence of alkyl-labeled fatty acids, namely Alk14, Alk16, Alk18, and Alk20, were employed to investigate this biological phenomenon. PPARγ can be efficiently labeled with palmitoylation (using Alk16) but shows significantly lower efficiency when labeled with Alk14, stearoylation (Alk18), or Alk20 (Supplementary figure 8A-C). This suggests that C16-captured S-palmitoylation is the primary acyl group involved in the chemical alteration of PPARγ. Having the C313 mutation as the critical site of PPARγ palmitoylation, we next evaluated the functional impact of these mutants on C16-catched S-palmitoylation over the duration of alkynyl palmitic acid (Alk16) therapy. In the CRC cells that were genetically modified, the presence of PPARγ with human C313S effectively eliminated the labeling of palmitoylation (with Alk16). This was confirmed by the streptavidin pull-down study, which showed that C313 was necessary for the S-palmitoylation of the PPARγ protein (Supplementary figure 8A-C). Furthermore, the role of ZDHHC6 as the primary target for PPARγ was established using a comparative study. This analysis demonstrated the anticipated changes in the quantity of PPARγ protein in HCT116 cells when ZDHHC6 was overexpressed using adenovirus, both with and without the PPARγ C313S mutation. This was confirmed by an immunofluorescence assay (Figure 6F). In addition, the absence of palmitoylation due to the C313S mutation resulted in a reduction in the simultaneous expression of PPARγ and pan-palmitoylation, as indicated in Figure 6F. Based on our previous findings, we have determined the interaction between ZDHHCs and their targeted substrates. The Co-IP test clearly showed a strong direct binding between ZDHHC6 and PPARγ in the ectopic expression of CRC cells (Figure 5). To enhance the visual representation of PPARγ palmitoylation in the presence of ZDHHC6, we employed Alk16 as a metabolic marker to assess the impact of ZDHHC6 on palmitoylated PPARγ and its movement inside cells, similar to what was shown in Supplementary figure 8. The presence of NH_2_OH significantly reduced the levels of palmitoylation on PPARγ, suggesting that the increase in palmitoylated PPARγ primarily occurred on cysteine residues and was caused by the action of ZDHHC6 (Figure 6G). Analysis of fractionation in ZDHHC6 wild-type or *ZDHHC6*-deficient HCT116 cells indicated that the palmitoylation of PPARγ by ZDHHC6 increased the quantity of the modified PPARγ protein specifically in the cell nucleus, but not in other cellular components (Figure 6H). The reliable outcomes of modifying the level of PPARγ through palmitoylation were further validated in HCT116 cells that overexpressed ZDHHC6 (Supplementary figure 8D). The aforementioned results consistently demonstrated that ZDHHC6 plays a crucial role as a primary palmitoyltransferase in the formation of palmitoylated PPARγ. Furthermore, its function exhibited a significant correlation with the advancement of colorectal cancer in human individuals.

**Upregulated ZDHHC6-mediated palmitoylated PPARγ promotes its nucleus translocalization**

Following the identification of ZDHHC6 as the primary palmitoyltransferase for PPARγ, the subsequent step was examining the impact of ZDHHC6 on the nucleus localization of PPARγ. This enzyme had an impact not only on the stabilization of PPARγ throughout the process of ZDHHC6 knockdown, but also on the palmitoylation of PPARγ (Figure 7A-C). In addition, the expression of ZDHHC6 by ectopic expression in HCT116, SNU-C2A, SW48, HT-29, and Caco-2 cells led to an increase in the expression of PPARγ protein and a speeding up of its translocation to the nucleus of the CRC cells (Figure 7D, E). The purpose of this investigation was to determine whether ZDHHC6 interacts with PPARγ in additional colorectal cancer cells. This was done in consideration of the determination of the interaction between DHHCs and their targeted substrates. As a matter of fact, the Co-IP test demonstrated that the direct interaction between ZDHHC6 and PPARγ could be wonderfully observed in the ectopic expression of human Caco-2 cells (Figure 7F). Furthermore, considering the fact that C313 site has been identified as the primary location for palmitoylation of PPARγ, we pose the question of whether or not the *S*-palmitoylation that was captured by C16 was the primary contributor to the chemical change of PPARγ. Consequently, to investigate this biological process, a series of alkyl-labeled fatty acylation operations, which included alk-C14, alk-C16, alk-C18, and alk-C20, were utilized. Palmitoylation (alk-C16) labels are able to successfully mark the PPARγ, whereas labels with alk-C14 chain lengths, stearoylation (alk-C18), or alk-C20 labels are significantly less effective in marking the PPARγ (Figure 7G, H). The findings of this study suggest that the primary acyl group responsible for the chemical alteration of PPARγ is C16-catched *S*-palmitoylation. After determining that the C313S site mutation is the most important location for PPARγ palmitoylation, we proceeded to investigate the functional effects of these mutations on C16-catched *S*-palmitoylation during the course of treatment with alkynyl palmitic acid (alk-C16). It was anticipated that PPARγ with a human C313S site mutation would drastically eliminate palmitoylation (alk-C16) labels, as corroborated by streptavidin pull-down analysis, which further demonstrated that human C313S was necessary for *S*-palmitoylation of PPARγ protein (Figure 7I). Moreover, to further visualize PPARγ palmitoylation in the presence of ZDHHC6, we utilized alk-C16 as a metabolic sign to investigate the effects of ZDHHC6 on palmitoylated PPARγ and its nucleus translocation. This was done in a manner that was comparable to the methodology described above. Incubation with NH_2_OH resulted in a significant reduction of palmitoylation levels on PPARγ, which suggests that the upregulation of palmitoylated PPARγ mostly happened on cysteine and was driven by ZDHHC6 (Figure 7J). An additional protocol was made by fractionation analysis in ZDHHC6 wild-type or *ZDHHC6*-deficient Caco-2 cells, which indicated that ZDHHC6-mediated palmitoylation of PPARγ increased the quantity of the modified PPARγ protein in the nucleus, but not in the membrane or cytoplasm components (Figure 7K). The studies presented above unambiguously demonstrated that ZDHHC6 is a significant palmitoyltransferase that plays a role in the occurrence of palmitoylated PPARγ, and the activity of this enzyme was found to have a favorable correlation with the progression of colorectal cancer.

**Palmitoylation-****stabilized PPARγ suppresses its** **lysosome degradation**

Prior report has demonstrated that the significant rise in PPARγ protein levels effectively halted the process of lysosome degradation in the face of prolonged metabolic challenges and stress ^36^. PPARγ is believed to undergo many posttranslational modifications that interact either positively or negatively to influence its protein destiny during different physiological and pathological states ^36, 37^. Therefore, we examined the impact of palmitoylated PPARγ on the evolution of its breakdown in the lysosome. The rapid breakdown of the palmitoylation-deficient mutant PPARγ C313S and the buildup of cellular lipids could be reversed by the lysosomal inhibitors NH_4_Cl and Pepstatin A (Pep A), but not by the proteasomal inhibitor MG132 or the autophagy inhibitor 3-Methyladenine (3-MA) (Figure 8A). In order to validate the lysosome-dependent mechanism, we utilized 2-BP to hinder the palmitoylation of naturally occurring PPARγ in HCT116 cells (Figure 8B). Pep A and NH_4_Cl, but not 3-MA or MG132, were able to enhance the stability of PPAR following depalmitoylation and a decrease in destabilized PPARγ-related lipid accumulation (Figure 8C, D).
 PPARγ, a nuclear receptor, has been found to have a significant impact on glucose metabolism and overall energy balance through its posttranslational alterations ^38^. Upon transportation to the cell nucleus, PPARγ can undergo various modifications such as phosphorylation, ubiquitination, and acetylation ^39, 40^. These modifications can affect its activity, stability, and interaction with other molecules. Additionally, PPARγ may be internalized into recycle endosomes or degraded through the late endosome-lysosome pathway ^41^. To investigate the impact of (de)palmitoylation on the movement of PPARγ, we inhibited the palmitoylation process of PPARγ and examined its distribution across various subcellular compartments. Consistent with expectations, the use of 2-BP significantly reduced the overlap of PPARγ with recycling endosomes indicated by Rab11, while increasing its overlap with lysosomes labeled by Lamp1 and late endosomes labeled by Rab7b (Figure 8E-G). The results consistently showed that depalmitoylation facilitated the degradation of PPARγ through the lysosomal pathway.

**ZDHHC6-mediated fatty acid biosynthesis promotes CRC carcinogenesis by upregulating** **PPARγ**

To ascertain the impact of increased production of fatty acids on cell proliferation, we manipulated the expression of PPARγ in cells that either had ectopically produced PPARγ or had been subjected to *ZDHHC6*-knockdown. In our study, we examined the process of glucose oxidation converting into fatty acid synthesis. We accomplished this by cultivating cells with glucose that was consistently enriched with carbon-13 ([U-^13^C] glucose). We specifically focused on cells that had reduced levels of PPARγ and overexpressed ZDHHC6. Our findings showed that when ZDHHC6 was overexpressed alone, it greatly enhanced the labeling of fatty acids from glucose tracers. However, when ZDHHC6 was overexpressed along with the co-transfection of shPPARγ, the knockdown of PPARγ significantly reduced the labeling of fatty acids from glucose tracers (Figure 9A). In contrast, the introduction of PPARγ-transduction greatly enhanced the process of labeling fatty acids from glucose tracers in HCT116 cells with ZDHHC6 knockdown (Figure 9B). Furthermore, the analysis of bodipy green staining and PPARγ co-expression revealed a consistent pattern of lipid buildup in the aforementioned CRC cells (Figure 9C, D). To further investigate the relationship between PPARγ and ZDHHC6, we manipulated the expression of PPARγ in cells lacking ZDHHC6 or cells overexpressing ZDHHC6 by co-transfecting them with a mutant form of PPARγ (PPARγ C313S). We conducted an experiment where we cultured cells with consistently labeled [U-^13^C] glucose in two different types of cells: *ZDHHC6*-knockout HCT116 cells and cells with ZDHHC6 restoration and Ad*PPARG* C313S mutant co-transfection. As expected, we observed glucose oxidation leading to fatty acid biosynthesis in both types of cells. However, when PPARγ C313S expression was present, it counteracted the increase in fatty acid synthesis regulated by ZDHHC6 and significantly reduced the labeling of fatty acids from glucose tracers (Supplementary figure 9A). In *ZDHHC6*-knockdown HCT116 cells, there is no significant detection of enhanced fatty acid labeling from glucose tracers when PPARγ C313S mutant-transduction is used (Supplementary figure 9B). Furthermore, the immunofluorescence assay revealed a consistent pattern of lipid accumulation in the aforementioned CRC cells, as demonstrated by the co-expression of PPARγ and the bodipy green staining (Supplementary figure 9C, D). These findings indicate that ZDHHC6 enhances the production of fatty acids by stabilizing PPARγ. To additionally confirm the role of ZDHHC6 in promoting tumor formation through the activation of fatty acid production, we performed xenograft tumor tests utilizing the aforementioned cell lines. Significantly, mice that received implantation of ZDHHC6-overexpressing HCT116 cells developed larger tumors in comparison to those implanted with control HCT116 cells. However, HCT116 cells that were simultaneously overexpressed with ZDHHC6 and co-transfected with sh*PPARγ* or PPARγ C313S mutants exhibited lower rates of tumor growth (Figure 9E; Supplementary figure 9E). In addition, mice that had been implanted with HCT116 cells in which ZDHHC6 had been suppressed and PPAR had been overexpressed exhibited higher rates of tumor formation compared to the CRC cells that had only been transfected with sh*ZDHHC6* (Figure 9F). In addition, the mice carrying the *ZDHHC6* knockdown and mutant PPARγ C313S HCT116 cells exhibited significantly suppressed tumor formation compared to the mice with shControl (Supplementary figure 9F). The results demonstrate that increased expression of ZDHHC6 can stimulate the production of fatty acids in living organisms by activating PPARγ, hence promoting the advancement of colorectal cancer.
 On top of that, we have found a strong positive correlation between ZDHHC6 and PPARγ, PPARγ and ALCY, and PPARγ and ACC transcript levels in the ICGC CRC and TCGA CRC databases. This correlation confirms the relationship between PPARγ and its associated factors during CRC formation (Figure 1 and Supplementary Figure 5). Subsequently, we assessed the predictive significance of ZDHHC6 and PPARγ in these datasets of colorectal cancer tissue microarrays (TMA). It is worth mentioning that patients with elevated levels of ZDHHC6 or PPARγ experienced significantly shorter overall survival compared to patients with low levels of ZDHHC6 or PPARγ in the TCGA CRC and ICGC CRC databases (Figure 9G, H). To better understand the impact of PPARγ on the prognosis of ZDHHC6-positive CRC patients, we conducted an analysis of the prognostic value of ZDHHC6 in four different CRC databases: CGWB, UCSC, CANEVOLVE, and COSMIC. This analysis included a total of 671 patients, with those who died within 5 months or were followed up for less than 3 months being excluded. Additionally, we examined the correlation between ZDHHC6 and various factors such as pathologic state (I–IV), race, gender, and age in the indicated CRC datasets (Supplementary figure 9G–K). CRC patients exhibiting a single elevated level of ZDHHC6 demonstrated an unfavorable prognosis. Also, individuals with elevated levels of ZDHHC6 have poorer overall survival compared to those with lower levels of this factor. Furthermore, there is a positive association between ZDHHC6 levels and pathological state but no significant correlation with race, age, or gender. Together, we discovered a previously unknown harmful connection between ZDHHC6 and the variables that regulate the production of fatty acids and the balance of lipid oxidation, specifically related to PPARγ, in patients with colorectal cancer.

**Discussion**

Disruption of cellular metabolism is a characteristic feature of the advancement of cancer. Alongside increased glycolysis, there are often abnormalities in fatty acid production and lipid oxidation in developing tumors, which are necessary to fulfill their metabolic needs ^42, 43^. Fatty acid production is less active in quiescent cells, which primarily take up lipids from the extracellular circulation. On the other hand, de novo lipogenesis (DNL), particularly the production of new fatty acids, plays a significant role in providing tumor cells with a source of lipids ^44^. In this study, we have discovered that ZDHHC6 functions as a palmitoyltransferase enzyme that controls the production of fatty acids. Specifically, ZDHHC6 directly adds palmitoyl groups to PPARγ, a protein involved in regulating gene expression. This palmitoylation process stabilizes PPARγ, leading to the activation of ACLY expression and the subsequent development of lipid buildup-related carcinogenesis (Figure 10).
 Alteration of the equilibrium in lipid oxidation and lipid synthesis is becoming a more significant contributor to the development of colorectal cancer (CRC) ^45^. This highlights the intricate relationship between cancer metabolism and cellular signaling pathways. The upregulation of enzymes like ACLY is responsible for this metabolic change, which not only supplies essential lipids for the formation of cell membranes but also enhances cancer-causing signaling pathways involved in cell survival, growth, and specialization ^46, 47^. Moreover, the production of particular lipid species can influence the activation of these pathways, so strengthening a cancer-promoting milieu. Elevated fatty acid synthesis also affects cellular energy production and can modify the tumor microenvironment, so promoting a favorable setting for cancer advancement and spread. Inhibiting this process of lipid production has demonstrated encouraging outcomes in impeding tumor growth and is a tempting target for therapeutic intervention ^48, 49^. Unraveling the exact ways in which fatty acid production and signaling pathways come together could reveal new possibilities for focused treatments in managing CRC ^50^. The latest findings align with prior studies, although they are even more noteworthy. This intensifies our curiosity about the sample set and motivates us to investigate the underlying factors.
 Aberrant expression or activation of associated enzymes leads to alterations in lipidome metabolic balance ^51^. Currently, there is significant research being conducted on the involvement of zinc finger-aspartate-histidine-cysteine (DHHC)-CRD-type palmitoyl acyltransferases (ZDHHCs) in the stabilization of oncoproteins and their impact on the advancement of cancer. Homo sapiens and Mus musculus encode a combined total of 23 ZDHHCs. Multiple lines of evidence suggest that ZDHHCs have crucial functions in the process of lipogenesis ^35, 52^. ZDHHC2 exhibits aberrant upregulation in renal cell carcinoma and plays a role in lipid production and carcinogenesis by modulating the ZDHHC2-AGK signaling axis ^53^. ZDHHC18 functions as a palmitoyltransferase for MDH2, promoting the formation of ovarian cancer by maintaining mitochondrial respiration and reinstating the growth and clonogenic potential of ovarian cancer cells ^54^. ZDHHC3 exacerbates the development of nonalcoholic steatohepatitis (NASH) and the progression of hepatocellular carcinoma (HCC) associated to NASH by boosting the accumulation of lipids regulated by IRHOM2 and the production of lipids mediated by FASN signaling ^35^. Furthermore, we have discovered a distinct variation in the expression of ZDHHC6 between colorectal cancer (CRC) and normal tissues. Notably, this difference in ZDHHC6 expression exhibits a substantial association with ACLY and the biological processes associated to lipid production. ZDHHC6 acts as an oncogenic protein in the process of tumor formation. It is significantly upregulated in various types of malignancies, including CRC. ZDHHC6 exhibits an oncogenic role in conjunction with AEG-1 under specific circumstances. ZDHHC6 has also been identified as a predictive gene in the human pathology atlas. Prior research has shown the varied impacts of ZDHHC6 on biological mechanisms linked to the advancement of cancer ^21^. ZDHHC6 plays a crucial function in cancer by exerting its impact on the growth and viability of cells. Evidence demonstrates that it stimulates the proliferation of cancerous cells by augmenting the functionality of growth factor receptors, such as epidermal growth factor receptor (EGFR) and platelet-derived growth factor receptor (PDGFR) ^55, 56^. Furthermore, ZDHHC6 plays a vital role in palmitoylating the PI3K/Akt pathway, which is essential for cellular longevity and the ability to fight apoptosis ^57, 58^. In addition, ZDHHC6 has the ability to regulate metastasis, which is a significant factor in cancer-related fatalities ^14, 59^. It enhances the invasive and migratory properties of cancer cells via controlling the production and function of matrix metalloproteinases (MMPs) and focal adhesion kinase (FAK). ZDHHC6 also participates in epithelial-mesenchymal transition (EMT), a biological mechanism linked to heightened metastatic capability. Moreover, ZDHHC6 has been discovered to influence angiogenesis, the process of creating new blood vessels that are necessary for the development of tumors ^58, 60^. It enhances the release of pro-angiogenic substances such as vascular endothelial growth factor (VEGF), which stimulates the growth and creation of blood vessels by increasing the number of endothelial cells. Nevertheless, our findings indicate that ZDHHC6 facilitates the production of fatty acids from scratch in colorectal cancer (CRC). Furthermore, the atypical buildup of lipids induced by ZDHHC6 was partially reliant on the heightened expression of ACLY. ACLY, a pivotal enzyme regulating the production of new lipids, enhances the progression of CRC. Furthermore, the heightened expression of ACLY enhances the production of fatty acids and stimulates the formation of tumors. Our investigation revealed that specifically reducing the activity of ACLY significantly suppressed the promotion of tumor growth and the enhancement of fatty acid production induced by overexpression of ZDHHC6.
 PPARγ is a crucial transcription factor that controls the production of lipids by increasing the transcription of enzymes involved in lipid synthesis, such as ACLY ^61^. It exhibits significant expression in adipocytes and plays a role in the absorption, production, and retention of lipids. In this study, we discovered that PPARγ plays a role in the upregulation of ACLY by ZDHHC6 in the nucleus. Additionally, we showed that the palmitoylation of PPARγ by ZDHHC6 is essential for stabilizing PPARγ and allowing its incorporation into the nucleus. Increased expression of PPARγ stimulates the production of lipids and the development of tumors, together with the activation of ACLY in colorectal cancer (CRC). Our work discovered that ZDHHC6 may function as a precursor to ACLY. Nevertheless, regardless of the presence or absence of ACLY, ZDHHC6 still enhances the expression of PPARγ. Thus, ZDHHC6 has a role in stabilizing PPARγ regardless of ACLY expression, indicating that ZDHHC6 is involved in numerous regulatory pathways. Furthermore, during transportation to the cell nucleus, PPARγ can undergo several posttranslational changes that can impact its activity, stability, and interaction with other molecules. ZDHHC6’s role in colon cancer lipid metabolism underscores a multifaceted regulatory mechanism that supports tumor growth and survival. Understanding the precise molecular interactions and pathways modulated by ZDHHC6-mediated palmitoylation will be essential for developing targeted therapies. Future research should focus on elucidating these mechanisms and exploring the therapeutic potential of ZDHHC6 inhibition in colon cancer treatment.

In summary, we demonstrate that ZDHHC6 stabilizes PPARγ through enhancing palmitoylation and greatly reducing its lysosomal degradation progress. The Cys-313 location at the DBD domain of PPARγ has been determined to be significant for the palmitoylation of PPARγ by ZDHHC6. PPARγ knockdown, in the meantime, eliminated the increase in ACLY expression and significantly reduced both carcinogenesis and fatty acid production induced by ZDHHC6 overexpression in CRC cells and xenograft tissues. Furthermore, patients who have colorectal cancer (CRC) and exhibit high expression levels of both ZDHHC6 and PPARγ tend to have an unfavorable prognosis and lower overall survival rates. To summarize, we have discovered a previously unknown signaling pathway called ZDHHC6-PPARγ pathway. This signaling is crucial in the process of lipid biosynthesis and CRC carcinogenesis. It also presents a potential target for cancer therapy that focuses on inhibiting fatty acid production.

**Ethics approval and consent to participate**

The use of clinical samples was approved by the ethics committee of Shandong First Medical University & Shandong Academy of Medical Sciences and informed consent were obtained from all patients (2023LC21074). All animal experimental protocols were performed following the Ethical Animal Care and Use Committee of Shandong University (2021sa0541jk).

**Consent for publication**

All authors consent to the publication of this article.

**Availability of data and material**

All data and material during the current study are available from the corresponding author on reasonable request.

**Competing interests**

The authors declare no competing interests.

**Funding**

This work was supported by (1) the Start-up fund of Shandong Cancer Hospital (NO. 2020PYA05); (2) the National Science Foundation of Shandong Province (NO. ZR2020MH254) and (3) the Science and Technology Program of Jinan (NO. 202134062).

**Author contributions**

Conceptualization & Methodology, Junqi Shan, Yanlai Sun;

Investigation, Junqi Shan, Xinyu Li, Runqi Sun, Yao Yao, Yan Sun, Qin Kuang, Xianling Dai, Yanlai Sun;

Data analysis, Junqi Shan, Xinyu Li, Runqi Sun, Yao Yao, Yanlai Sun;

Funding acquisition, Project administration & Supervision, Yanlai Sun;

Writing-original draft, Junqi Shan, Xinyu Li, Runqi Sun, Yanlai Sun;

Writing review & editing, Junqi Shan, Xinyu Li, Yanlai Sun.

All authors have read and approved the final manuscript.

**Abbreviations**

CRC: Colorectal cancer

ZDHHC6: Zinc finger DHHC-type palmitoyltransferase 6

ATCC: American Type Culture Collection

PPARγ: Peroxisome proliferator-activated receptor gamma

FBS: Fetal bovine serum

IHC: Immunohistochemistry

TCGA: The cancer genome database

1. BP: 2-bromopalmitate

HA: Hyaluronic acid

CM: Cytomembrane

ACLY: ATP citrate lyase

SCD: Stearoyl-CoA desaturase

FASN: Fatty acid synthase

ICGC: International cancer genome consortium

GEO: Gene expression omnibus

**References**

1. Siegel, Rebecca L., et al. "Colorectal cancer statistics, 2023." *CA: a cancer journal for clinicians* 73.3 (2023): 233-254.
2. Morgan, Eileen, et al. "Global burden of colorectal cancer in 2020 and 2040: Incidence and mortality estimates from GLOBOCAN." *Gut* 72.2 (2023): 338-344.
3. Nadeem, Muhammad Shahid, et al. "Risk of colorectal cancer in inflammatory bowel diseases." *Seminars in cancer biology*. Vol. 64. Academic Press, 2020.
4. Bui, Triet M., et al. "Tissue-specific reprogramming leads to angiogenic neutrophil specialization and tumor vascularization in colorectal cancer." *The Journal of Clinical Investigation* (2024).
5. Sanford, Nina N., et al. "Obesity and younger versus older onset colorectal cancer in the United States, 1998–2017." *Journal of gastrointestinal oncology* 11.1 (2020): 121.
6. Murphy, Caitlin C., et al. "Maternal obesity, pregnancy weight gain, and birth weight and risk of colorectal cancer." *Gut* 71.7 (2022): 1332-1339.
7. Ecker, Josef, et al. "The colorectal cancer lipidome: identification of a robust tumor-specific lipid species signature." *Gastroenterology* 161.3 (2021): 910-923.
8. Wasinger, Valerie C., et al. "Spp24 is associated with endocytic signalling, lipid metabolism, and discrimination of tissue integrity for ‘leaky-gut’in inflammatory bowel disease." *Scientific reports* 10.1 (2020): 12932.
9. Sclafani, Francesco, et al. "Analysis of KRAS, NRAS, BRAF, PIK3CA and TP53 mutations in a large prospective series of locally advanced rectal cancer patients." *International Journal of Cancer* 146.1 (2020): 94-102.
10. Martinelli, E., et al. "Implementing anti-epidermal growth factor receptor (EGFR) therapy in metastatic colorectal cancer: challenges and future perspectives." *Annals of Oncology* 31.1 (2020): 30-40.
11. Li, Wen, et al. "Insights into the post-translational modification and its emerging role in shaping the tumor microenvironment." *Signal Transduction and Targeted Therapy* 6.1 (2021): 422.
12. Lin, Xiangyi, et al. "Advances of Protein Palmitoylation in Tumor Cell Deaths." *Cancers* 15.23 (2023): 5503.
13. Bartolacci, Caterina, et al. "Lipid metabolism regulates oxidative stress and ferroptosis in RAS-driven cancers: a perspective on cancer progression and therapy." *Frontiers in molecular biosciences* 8 (2021): 706650.
14. Ko, Pin‐Joe, and Scott J. Dixon. "Protein palmitoylation and cancer." *EMBO reports* 19.10 (2018): e46666.
15. Greaves, Jennifer, and Luke H. Chamberlain. "DHHC palmitoyl transferases: substrate interactions and (patho) physiology." *Trends in biochemical sciences* 36.5 (2011): 245-253.
16. Stix, Robyn, et al. "Structure and mechanism of DHHC protein acyltransferases." *Journal of molecular biology* 432.18 (2020): 4983-4998.
17. Gao, Tian, et al. "Recent progress of palmitoyl transferase DHHC3 as a novel antitumor target." *Future Medicinal Chemistry* 14.6 (2022): 443-455.
18. Salaun, Christine, Nicholas CO Tomkinson, and Luke H. Chamberlain. "The endoplasmic reticulum–localized enzyme zDHHC6 mediates S-acylation of short transmembrane constructs from multiple type I and II membrane proteins." *Journal of Biological Chemistry* 299.10 (2023).
19. Fhu, Chee Wai, and Azhar Ali. "Protein lipidation by palmitoylation and myristoylation in cancer." *Frontiers in Cell and Developmental Biology* 9 (2021): 673647.
20. Zhang, Yun, et al. "Oxidized high-density lipoprotein promotes CD36 palmitoylation and increases lipid uptake in macrophages." *Journal of Biological Chemistry* 298.6 (2022).
21. Zhou, Binhui, et al. "The palmitoylation of AEG-1 dynamically modulates the progression of hepatocellular carcinoma." *Theranostics* 12.16 (2022): 6898.
22. Fredericks, Gregory J., et al. "Stable expression and function of the inositol 1, 4, 5-triphosphate receptor requires palmitoylation by a DHHC6/selenoprotein K complex." *Proceedings of the National Academy of Sciences* 111.46 (2014): 16478-16483.
23. Marciel, Michael P., and Peter R. Hoffmann. "Molecular mechanisms by which selenoprotein K regulates immunity and cancer." *Biological Trace Element Research* 192 (2019): 60-68.
24. Pagano, Cristina, et al. "Advances in “adiponcosis”: Insights in the inner mechanisms at the base of adipose and tumour tissues interplay." *International Journal of Cancer* 152.12 (2023): 2464-2473.
25. Wagner, Nicole, and Kay-Dietrich Wagner. "PPAR beta/delta and the hallmarks of cancer." *Cells* 9.5 (2020): 1133.
26. Cheng, Hong Sheng, et al. "PPARs and tumor microenvironment: the emerging roles of the metabolic master regulators in tumor stromal–epithelial crosstalk and carcinogenesis." *Cancers* 13.9 (2021): 2153.
27. Morigny, Pauline, et al. "Lipid and glucose metabolism in white adipocytes: pathways, dysfunction and therapeutics." *Nature Reviews Endocrinology* 17.5 (2021): 276-295.
28. Faghfouri, Amir Hossein, et al. "PPAR-gamma agonists: Potential modulators of autophagy in obesity." *European journal of pharmacology* 912 (2021): 174562.
29. Salita, Timothy, et al. "Reprogrammed lipid metabolism and the lipid-associated hallmarks of colorectal cancer." *Cancers* 14.15 (2022): 3714.
30. Pan, Shijia, et al. "Therapeutic potential of melatonin in colorectal cancer: Focus on lipid metabolism and gut microbiota." *Biochimica et Biophysica Acta (BBA)-Molecular Basis of Disease* 1868.1 (2022): 166281.
31. Jin, Xin, et al. "Pathophysiology of obesity and its associated diseases." *Acta Pharmaceutica Sinica B* (2023).
32. Wang, Dingzhi, et al. "Crosstalk between peroxisome proliferator-activated receptor δ and VEGF stimulates cancer progression." *Proceedings of the National Academy of Sciences* 103.50 (2006): 19069-19074.
33. You, Mengli, et al. "PPARδ signaling regulates colorectal cancer." *Current pharmaceutical design* 21.21 (2015): 2956-2959.
34. Lin, Mao Song, et al. "Expression of PPARγ and PTEN in human colorectal cancer: An immunohistochemical study using tissue microarray methodology." *Oncology letters* 2.6 (2011): 1219-1224.
35. Xu, Minxuan, et al. "Palmitoyltransferase ZDHHC3 Aggravates Nonalcoholic Steatohepatitis by Targeting S‐Palmitoylated IRHOM2." *Advanced Science* 10.28 (2023): 2302130.
36. Tan, Hayden Weng Siong, et al. "Lysosomal inhibition attenuates peroxisomal gene transcription via suppression of PPARA and PPARGC1A levels." *Autophagy* 15.8 (2019): 1455-1459.
37. Videira, Natália B., et al. "PPAR modulation through posttranslational modification control." *Nuclear Receptors: The Art and Science of Modulator Design and Discovery* (2021): 537-611.
38. Scholtes, Charlotte, and Vincent Giguère. "Transcriptional control of energy metabolism by nuclear receptors." *Nature Reviews Molecular Cell Biology* 23.11 (2022): 750-770.
39. Wadosky, Kristine M., and Monte S. Willis. "The story so far: post-translational regulation of peroxisome proliferator-activated receptors by ubiquitination and SUMOylation." *American Journal of Physiology-Heart and Circulatory Physiology* 302.3 (2012): H515-H526.
40. Porcuna, Jesús, Jorge Mínguez-Martínez, and Mercedes Ricote. "The PPARα and PPARγ epigenetic landscape in cancer and immune and metabolic disorders." *International journal of molecular sciences* 22.19 (2021): 10573.
41. Chinetti-Gbaguidi, G., et al. "Peroxisome proliferator-activated receptor α controls cellular cholesterol trafficking in macrophages." *Journal of lipid research* 46.12 (2005): 2717-2725.
42. Chen, D. et al. A Multidimensional Characterization of E3 Ubiquitin Ligase and Substrate Interaction. Netw. iScience 16, 177–191 (2019).
43. Visweswaran, Malini, et al. "Aberrant lipid metabolism as an emerging therapeutic strategy to target cancer stem cells." *Stem cells* 38.1 (2020): 6-14.
44. Mahmud, Iqbal, et al. "DAXX drives de novo lipogenesis and contributes to tumorigenesis." *Nature communications* 14.1 (2023): 1927.
45. Mozolewska, Paulina, et al. "Inhibitors of fatty acid synthesis and oxidation as potential anticancer agents in colorectal cancer treatment." *Anticancer Research* 40.9 (2020): 4843-4856.
46. Qiao, Chenyang, et al. "IGF1-mediated HOXA13 overexpression promotes colorectal cancer metastasis through upregulating ACLY and IGF1R." *Cell Death & Disease* 12.6 (2021): 564.
47. Schulz-Kuhnt, Anja, et al. "ATP citrate lyase (ACLY)-dependent immunometabolism in mucosal T cells drives experimental colitis in vivo." *Gut* (2024).
48. Broadfield, Lindsay A., et al. "Lipid metabolism in cancer: New perspectives and emerging mechanisms." *Developmental Cell* 56.10 (2021): 1363-1393.
49. Beloribi-Djefaflia, S., S. Vasseur, and F. Guillaumond. "Lipid metabolic reprogramming in cancer cells." *Oncogenesis* 5.1 (2016): e189-e189.
50. Krauß, Dana, Ourania Fari, and Maria Sibilia. "Lipid Metabolism Interplay in CRC—An Update." *Metabolites* 12.3 (2022): 213.
51. Fernández, Lara P., Marta Gomez de Cedron, and Ana Ramirez de Molina. "Alterations of lipid metabolism in cancer: implications in prognosis and treatment." *Frontiers in oncology* 10 (2020): 577420.
52. Wang, Juan, et al. "DHHC4 and DHHC5 facilitate fatty acid uptake by palmitoylating and targeting CD36 to the plasma membrane." *Cell reports* 26.1 (2019): 209-221.
53. Sun, Yan, et al. "ZDHHC2-Mediated AGK Palmitoylation Activates AKT–mTOR Signaling to Reduce Sunitinib Sensitivity in Renal Cell Carcinoma." *Cancer Research* 83.12 (2023): 2034-2051.
54. Pei, Xuan, et al. "Palmitoylation of MDH2 by ZDHHC18 activates mitochondrial respiration and accelerates ovarian cancer growth." *Science China Life Sciences* 65.10 (2022): 2017-2030.
55. Coronel Arrechea, Consuelo, et al. "A novel yeast-based high-throughput method for the identification of protein palmitoylation inhibitors." *Open Biology* 11.8 (2021): 200415.
56. Tecik, Melisa, and Aysun Adan. "Therapeutic targeting of FLT3 in acute myeloid leukemia: current status and novel approaches." *OncoTargets and Therapy* (2022): 1449-1478.
57. Lin, Hening. "Protein cysteine palmitoylation in immunity and inflammation." *The FEBS journal* 288.24 (2021): 7043-7059.
58. Zhou, Binhui, et al. "Protein palmitoylation in cancer: molecular functions and therapeutic potential." *Molecular oncology* 17.1 (2023): 3-26.
59. Qu, Mengyuan, et al. "Lipid-induced S-palmitoylation as a vital regulator of cell signaling and disease development." *International journal of biological sciences* 17.15 (2021): 4223.
60. Wu, Zeang, et al. "Protein S-Palmitoylation and Lung Diseases." *Lung Inflammation in Health and Disease, Volume II*. Cham: Springer International Publishing, 2021. 165-186.
61. Noh, Kyung Hee, et al. "Ubiquitination of PPAR-gamma by pVHL inhibits ACLY expression and lipid metabolism, is implicated in tumor progression." *Metabolism* 110 (2020): 154302.
