# Supplementary material for: Palmitoyltransferase ZDHHC6 promotes colon tumorigenesis by targeting PPARγ-driven lipid biosynthesis via regulating lipidome metabolic reprogramming
Source: J Exp Clin Cancer Res. 2024 Aug 16;43:227. doi: 10.1186/s13046-024-03154-0 (PMC11328492; doi:10.1186/s13046-024-03154-0)
Supplement: Supplementary file 7 — Supplementary Material 7 [file 13046_2024_3154_MOESM7_ESM.docx]

**Response to the Reviewer Comments**

According to comments and suggestions, we improved this study. All changes made in the manuscript are marked as **red**.

**Reviewer #1:**
*The authors investigate the involvement of ZDHHC6 in the progression of CRC. They find that ZDHHC6 regulates lipid synthesis via PPARy-ACLY signalling and that its inhibition may represent a therapeutic target. Specifically, they find that ZDHHC6 adds palmitol groups to the Cys 313 site of PPARy, stabilising the protein and causing activation of the ACLY-associated metabolic pathway. Palmitolation inhibits liposome-induced degradation of PPARy by facilitating its translocation into the nucleus. The authors also note that inhibition of ZDHHC6 blocks growth in vivo. The work is not well written and the results shown do not explain what is reported in the text. Materials and methods are lacking, especially with regard to particular techniques such as IPs. Over-expression experiments are shown that distort somewhat the physiological condition. The proteins discussed are a transcription factor and a transmembrane reticulum protein. The authors do not show experiments in which demonstrate their colocalisation.*

We appreciate the reviewer’s comments on our manuscript’. Here we have worked hard to address all of the comments made by this reviewer.

*In general, there are errors in the figures.
Fig 5 E: I**n the bottom panel there was a reversal in one of the WB strips. In fact, in the first column the over-expression of Myc-ZDHH6 is indicated, but not FLAG-PPARg. In fact, only the former and not the latter is present in the input. In the IP, however, the bands of both appear and this is not possible.
More generally, the IP never shows an enrichment compared to the input and in some cases there is even a decrease.* *In Materials and Methods the technique is not* *adequately explained, the composition of the lysis buffers and the procedure is not indicated.*

Thank you very much for your concerns above. According to your comments, a series of different transfected human colon cancer cell lines (HCT116, Caco-2, SNU-C2A, and HT-29) was used to re-confirm the direct protein binding of ZDHHC6 and PPARγ and confirm the findings we found in our first version.

To demonstrate the specific target of ZDHHC6 responsible for its specific function, we performed an additional immunoprecipitation-MS (IP-MS) analysis of Flag-ZDHHC6 and His-PPARγ-transfected HCT116, Caco-2, SNU-C2A, and HT-29, respectively, and identified 126 detectable potential ZDHHC6-interacting proteins. We then integrated the ZDHHC6-interacting proteins obtained from the IP-MS experiment and the upregulated proteins revealed by proteomics analysis, and we identified PPARγ as the key protein that interacts with and is regulated by ZDHHC6 (revised Figures 5E, indicated below). Thus, PPARγ was selected as a candidate target of ZDHHC6.

On the other hand, Flag-ZDHHC6 and His-PPARγ were overexpressed in Caco-2, and SNU-C2A, respectively. Co-immunoprecipitation (Co-IP) experiments demonstrated that ZDHHC6 co-immunoprecipitated with PPARγ, and vice versa (revised Figures 5F, indicated below). Glutathione *S*-transferase (GST) pull-down assay suggested that ZDHHC6 interacts directly with PPARγ (revised Figures 5G, indicated below). This GST pull-down result is consistent with the direct protein interaction of ZDHHC6 and PPARγ in our first version.

(revised Figures 5E)

(revised Figures 5F and G)

More importantly, we have added detailed experimental materials and methods regarding the IP and GST pull-down assays in the M&M section to make our study easier to read and understand.

**Immunoprecipitation and S-Protein pull down assay**

Immunoprecipitation and SFB pull-down experiment was performed as described previously (Netw. iScience 16, 177–191 (2019)). Briefly, cells were lysed in E1A lysis buffer (250 mM NaCl, 50 mM HEPES [pH 7.5], 0.1% NP-40, 5 mM EDTA, protease inhibitor cocktail [Sigma]). The antibodie to ZDHHC6 was used for immunoprecipitation. HCT116 cells were transfected with SFB-tagged protein and lysed in NETN buffer (200 mM Tris-HCl [pH 8.0], 100 mM NaCl, 0.05% NP-40, 1 mM EDTA, protease inhibitor cocktail [Sigma]) for 20 min at 4 °C. Crude lysates were subjected to centrifugation at 14,000 × g for 15 min at 4 °C. Supernatants were incubated with S-Protein Agarose for 4 h (Millipore, USA). The agaroses were washed three times with NETN buffer. Proteins were eluted by boiling in 1× SDS loading buffer and subjected to SDS-PAGE for immunoblotting.

**GST pull-down assay**

GST pull-down assay was used to detect the direct interaction between PPARγ and ZDHHC6. Briefly, GST-tagged PPARγ (GST-PPARγ) and 6×His-tagged ZDHHC6 (His-ZDHHC6) or 3×Flag-tagged ZDHHC6 (Flag-ZDHHC6) proteins were expressed in BL21 (DE3) Escherichia coli via transforming pGEX-4T-1-GST-PPARγ and pET24a-His-ZDHHC6 or pET24a-Flag-ZDHHC6 plasmids, respectively. Then, the E. coli were collected, sonicated, and purified with cOmplete His-Tag Purification Resin (Roche) or Purification of Flag kit (Sigma-Aldrich) to obtain purified ZDHHC6-tagged protein. GST-PPARγ protein was expressed and immobilized with BeyoGold™ GST-tag Purification Resin (Beyotime) following the manufacturer’s instructions. The beads-PPARγ complexes were washed with GST pull-down binding buffer (50 mM Tris-HCl, 200 mM NaCl, 1 mM EDTA, 1% NP-40, 1 mM DTT, 10 mM MyCl2, pH 8.0) and incubated with purified ZDHHC6-tagged protein at 4 °C for 4 h on a rotating windmill. Finally, the beads were washed and analyzed by immunoblotting assay.

*Ref: Chen, D. et al. A Multidimensional Characterization of E3 Ubiquitin Ligase and Substrate Interaction. Netw. iScience 16, 177–191 (2019).*

*Figure 5F: ZDHH6 and PPARg appear to have a completely opposite localisation. They do not colocalise.*

Thank you so much for your comments here. As we found and mentioned in our first version, ZDHHC6 is mainly localized in the cytoplasm and performs its catalytic function, while ZDHHC6-induced increased palmitoylation of PPARγ will be largely translocated to the nucleus and remain stable. Thus, in CRC cells with high lipid anabolism, abundant palmitoylated PPARγ expression was significantly observed in the nucleus but not in the cytoplasm. Also, our Click-iT and IP assays further indicated the consistent results we found above.

*Fig 6E: control without CHX is missing and the quality of the WB is not adequate.*

According to your suggestions here, we re-performed this experiment to further determine the protein abundance changes of PPARG *in vitro*. Also, DMSO was used as a control in the additional assay.

Consistent with the results we obtained in our first version, PPARG with the C313S mutant markedly decreased its intracellular protein stabilization, thereby resulting in a reduction in total PPARG protein abundance (revised Figure 6E).

(revised Figure 6E)

*Fig 6F: is described in the text as an immunoblot, but is an IF. Also,* *they theoretically show how mutated PPARg cannot be palmitoylated, but the anti-pamitoylation antibody should show all pamitoylation in the cell.* *Is it possible that mutating a residue on a target protein causes such a strong and generalised decrease in palmitoylation?*

Thank you very much for your kindly reminder. We are sorry for this incorrect statement in our manuscript. Thus, we have corrected this section as “This was confirmed by an immunofluorescence assay (Figure 6F)”.

Also, given the significant role of palmitoylation in the control of PPARγ stability, we were compelled to determine the precise position of palmitoylation on PPARγ. The function investigation involved determining the anticipated location of the palmitoylation site on PPARγ for *Homo sapiens* and *Mus musculus*. This was achieved through a combined analysis of the GPS-Palm software (MacOS_20200219) developed by The CUCKOO Workgroup (http://gpspalm.biocuckoo.cn/) and the MDD-Palm algorithm (http://csb.cse.yzu.edu.tw/MDDPalm/). Both methods fortuitously anticipated and furnished the foremost 4 palmitoylation sites for PPARγ, exhibiting distinct confidence intervals and quality ratings. It is worth mentioning that cysteine 313 (C313) in human PPARγ and mouse PPARγ were identified as the most probable and dependable location for protein palmitoylation modification.

Of note, because all cysteine sites are potential palmitoylation modification sites, as reported previously (Refs 1 and 2, see below), our current study did determine that the C313 mutant is a major site of palmitoylation for PPARγ. Thus PPARγ with C313 mutant did largely reduce ZDHHC6-mediated palmitoylation levels when we used anti-pan-pamitoylation antibody to detect its pamitoylation expression of PPARγ. Indeed, as we cited in the original literature for the antibody produce process and other colleagues of ours have used proven pan-pamitoylation antibodies (Refs 3 and 4; see below), as you mentioned above, we synchronously detected the PPARγ expression alterations when we used pan-pamitoylation antibodies in the IF assay. Also, on top of this, western blotting, Click-iT, and other biochemical assays were used simultaneously to support our results. These integrated results indicated that mutated PPARγ did decrease ZDHHC6-mediated pamitoylation modifications in vitro. PPARγ-regulated cell energy metabolism is essential for the functional performance of colon cancer cells (Refs 5 and 6; see below). Dysfunctional or mutant PPARγ protein expression, at least, directly or indirectly affected the balance of physiological states in cancer cells. These above reasons may synergistically result in a strong and generalized decrease in palmitoylation.

*Ref 1: Lin H. Protein cysteine palmitoylation in immunity and inflammation[J]. The FEBS journal, 2021, 288(24): 7043-7059.*

*Ref 2: Ko P J, Dixon S J. Protein palmitoylation and cancer[J]. EMBO reports, 2018, 19(10): e46666.*

*Ref 3: Yao H, Lan J, Li C, et al. Inhibiting PD-L1 palmitoylation enhances T-cell immune responses against tumours[J]. Nature biomedical engineering, 2019, 3(4): 306-317.*

*Ref 4: Xu M, Tan J, Zhu L, et al. Palmitoyltransferase ZDHHC3 Aggravates Nonalcoholic Steatohepatitis by Targeting S‐Palmitoylated IRHOM2[J]. Advanced Science, 2023, 10(28): 2302130.*

*Ref 5: Glazer R I, Yuan H, Xie Z, et al. PPARγ and PPARδ as modulators of neoplasia and cell fate[J]. PPAR research, 2008, 2008(1): 247379.*

*Ref 6: Wagner N, Wagner K D. Peroxisome proliferator-activated receptors and the hallmarks of cancer[J]. Cells, 2022, 11(15): 2432.*

*Fig 6H, shows that palmitoylated PPARG accumulates more in the nucleus than in the cytosol. In the IFs in 6A and 6F, however, there is no colocalisation between pan-palmitoylation and PPARg in the nucleus. There is a discrepancy.*

Thank you very much for your concerns in this point. As we responded above, ZDHHC6 is primarily found in the cytoplasm where it carries out its catalytic activity. On the other hand, enhanced palmitoylation of PPARγ caused by ZDHHC6 will mostly migrate to the nucleus where it will remain stable. Thus, substantial palmitoylated PPARγ expression was notably seen in the nucleus but not in the cytoplasm in CRC cells with strong lipid anabolism.

Actually, colocalisation between pan-palmitoylation and PPARγ in the nucleus was markedly confirmed in Figure 6A and 6F. According to your suggestion, the possible reason for the lack of obvious colocalization may be that the quality of the images we took was not adequate enough and the representative pictures were not significant enough. Therefore, we re-performed the experiment and the results obtained were consistent with the results of our first version and those found by other detection methods.

As expected, intracellular palmitoylation levels are consistent with PPARγ protein expression abundance in the cell nucleus. Meanwhile, suppression of palmitoylation by 2-BP or PPARγ with the C313 mutant did largely reduce its protein stabilization and total protein abundance in vitro experiments. The results of this part of additional repeated verification are consistent with the experimental results obtained by our other methods and mutually corroborate.

(revised Figure 6A)

(revised Figure 6F)

*In Figure 7E, the fluorescence intensity in the control is much lower than in the IFs in the other figures. In contrast, when ZDHHC6 is over-expressed, the fluorescence of PPARG is strong, saturated and present in the cytoplasm.*

According to your comments and as you mentioned the key point above, we further repeated and re-confirmed this experiment (revised Figure 7E, indicated below). Consistent with our other findings, CRC cells with ZDHHC6 overexpression did promote PPARγ expression stabilization and nucleus translocation.

(revised Figure 7E)

*In any case, it would be important to co-express ZDHHC6 and mutated PPARg in order to demonstrate that overexpression of ZDHHC6 is not able to stabilise and increase mutated PPARg protein levels.*

We fully agree with your comment here. Thus, we used HCT116 cells with *ZDHHC6* and *PPARG* double knockouts (*ZDHHC6-PPARG*) to confirm this experiment (indicated below). Accordingly, HCT116 (*ZDHHC6-PPARG* DKO) was expressed with PPARG (C313S) or co-expressed with PPARG (C313S) and ZDHHC6. Consistent with our other results, PPARG (C313S) expression did not significantly lead to its nucleus translocation. On the other hand, overexpressing ZDHHC6 does not significantly increase PPARG abundance and nucleus translocation.

*Furthermore, in Figure 7D (the WB) they show the HA tag to demonstrate that ZDHHC6 is over-expressed, but we do not know how much protein is actually expressed. It is not necessarily true that if there is the tag there is more protein than basal.*

According to your comments here, We re-checked the ZDHHC6 protein expression profiles in control (HA-ZDHHC6(-)) and ZDHHC6 vector-transfected cells (HA-ZDHHC6(+)) using the same cell lysates.

*The results shown in Figure 6H and 7K should be equivalent (same experiment). In fact, in the former case there is a lot of PPARg in the membrane and cytosol fractions and little in the nuclear. In 7K, on the other hand, there is a lot of PPARg in the cytosol and nuclear and not in the membrane fraction. The authors have to explain this fact. Furthermore, as PPARg is a soluble protein and a transcription factor, it should not be present in the membrane fraction. The error is therefore probably in Figure 6H. Furthermore, the level of ZDHHC6 and thus the silencing efficiency and localisation of the protein in relation to* *PPARg is never shown in the input.*

Thank you very much for your comments above. We fully agreed with your suggestion here, and this issue did catch our attention. Therefore, we re-examined our original data and concluded that the immunoblotting we presented in Figure 6H of the first version might have been caused by nonspecific binding or sample overflow during sample loading. Hence, to further confirm this key point, an additional experiment using the new Nuclear/Cytosol Extraction Kit (ab289882, Abcam) and PPARG antibody (NBP2-22106, Novus Biologicals) on new cell samples with the same experimental performance protocol was conducted. Also, based on the same experimental kits and protocols, in order to best confirm our results, the SNU-C2A cell (revised Figure 6H) and HCT116 cell (revised Figure 7K) were used to perform this experiment, respectively.

(revised Figure 6H)

(revised Figure 7K)

Consistent with our other findings and other Click-iT analysis, increased Alk16-tagged PPARG (increase in palmitoylation) by ZDHHC6 promoted its translocation to the cell nucleus. This re-confirmed result has been updated in our revised figures.

*Figure 7A shows that the silencing of ZDHHC6 is not 100% efficient and that some expression of PPARg also remains. In 7C, on the other hand, PPARg disappears when ZDHHC6 is silenced, there is no trace of how efficient ZDHHC6 was, and the palmitoylation signal (which I interpret as total protein palmitoylation) also disappears. It is not an IP, so it is not possible to speak of specific palmitoylation of PPARg, at the same time, ZDHHC6 is not the only palmitoylase in existence, so it is not credible that general palmitoylation of proteins disappears by silencing it.*

Thank you so much for these kind concerns. We used the colon cancer cells with ZDHHC6 gene knockout cell line in addition to the original cell line to better confirm the effect of ZDHHC6 on PPARG expression.

As expected in re-performed figures 7A and 7C, consistent with our previous findings and other results, colon cancer cells with ZDHHC6 deficiency did significantly reduce PPARγ protein expression, stabilization, and palmitoylation modification.

(revised Figure 7A)

(revised Figure 7C)

*Authors should include expression data of proteins investigated on human tissues of different stages in histology.*

Thank you very much for your comments here. According to your points, we additionally detected the ZDHHC6 protein expression in our in-house CRC patients (stages I–IV) samples that were randomly selected from the database. As expected, ZDHHC6 protein levels in CRC samples gradually increased with the progression of development (indicated below). Meanwhile, previous reports have indicated that with the development of CRC progression, PPARG activation promotes the proliferation of colorectal cancer cells and tumor growth (Ref 1 and 2; indicated below). Indeed, increased PPARG expression in our CRC samples is consistent with upregulated ZDHHC6 expression levels. On the other hand, examination of ZDHHC6 and PPARG expression in the TCGA public database further indicated consistent results.

*Ref 1. Schöckel L, Woischke C, Surendran S A, et al. PPARG activation promotes the proliferation of colorectal cancer cell lines and enhances the antiproliferative effect of 5-fluorouracil[J]. BMC cancer, 2024, 24(1): 234.*

*Ref 2. Girnun G. PPARG: a new independent marker for colorectal cancer survival[J]. Gastroenterology, 2009, 136(4): 1157-1160.*

**Reviewer #2:**

*In this manuscript, the authors investigated the potential role of ZDHHC6 on the progression of CRC and the possible functional correlation between ZDHHC6 and PPARγ stabilization. In the first part of the manuscript, a bioinformatic approach is performed to identify a potential list of candidate genes. Among these, ZDHHC6. Using TCGA data, patients with a high ZDHHC6 expression level had a noticeably worse survival prognosis. TCGA data was validated using 73 pairs of CRC samples and their matching adjacent samples. Moreover, the authors demonstrated that ZDHHC6 enhances lipid deposition and carcinogenesis in CRC cells through the regulation of PPARγ. Overall,* *data support the authors' conclusions. Figures are self-explanatory.
 Here some minor weaknesses to be addressed:*

We appreciate the reviewer’s compliment on our manuscript’s importance that “*data support the authors' conclusions. Figures are self-explanatory.*” We have worked hard to address all of the comments made by this reviewer.

*This demonstrated the significance of ZDHHC6 for the development of CRC. This demonstrated that ZDHHC6 is potentially associated with biological processes....*

Thank you so much for your comments here. We have re-checked the whole manuscript and re-corrected the description of some statements to ensure that the results and conclusions regarding our current study are more precise and accurate.

*In order to confirm the increased expression of ZDHHC6 in colorectal cancer (CRC), we obtained 73 pairs of CRC samples.... Is there a correlation between ZDHHC6 and the clinical data of these patients? Did the authors perform an IHC analysis of ZDHHC6 expression (in the human protein atlas database ZDHHC6 expression is "not detected")?*

Thank you very much for your comments above. We fully agreed with your suggestion here, and this issue did catch our attention. Thus, the corresponding pearson correlation analysis was performed to show the correlation of ZDHHC6 with CRC-related indicators (CA50%, CA125%, CA724%, CA199%, CA242%, CEA%, HCG% and CK-BB%) in patient samples (indicated below). Indeed, increased ZDHHC6 expression in samples was significantly observed with the up-regulation of CA50%, CA125%, CA724%, CA199%, CA242%, CEA%, HCG% and CK-BB%.

On the other hand, we additionally detected the ZDHHC6 protein expression in our in-house CRC patients (stages I–IV) samples that were randomly selected from the database. As expected, ZDHHC6 protein levels in CRC samples gradually increased with the progression of development (indicated below). Meanwhile, previous reports have indicated that with the development of CRC progression, PPARG activation promotes the proliferation of colorectal cancer cells and tumor growth (Ref 1 and 2; indicated below). Indeed, increased PPARG expression in our CRC samples is consistent with upregulated ZDHHC6 expression levels. On the other hand, examination of ZDHHC6 and PPARG expression in the TCGA public database further indicated consistent results.

*Ref 1. Schöckel L, Woischke C, Surendran S A, et al. PPARG activation promotes the proliferation of colorectal cancer cell lines and enhances the antiproliferative effect of 5-fluorouracil[J]. BMC cancer, 2024, 24(1): 234.*

*Ref 2. Girnun G. PPARG: a new independent marker for colorectal cancer survival[J]. Gastroenterology, 2009, 136(4): 1157-1160.*

*This finding was further validated through western blotting and immunofluorescence detection. Did the authors investigate ZDHHC6 cellular localization?*

Thank you very much for your concerns here. Previous reports have indicated the localization of ZDHHC6 to ER membranes by lysine-based sorting signals (Refs. 1 and 2, see below).

Furthermore, we found that the palmitoylation of PPARG primarily occurred in the cytoplasm, thereby promoting its nucleus translocation, and subsequent immunofluorescence analysis also confirmed that ZDHHC6 was mainly localized in the cytoplasm (indicated below).

*Ref 1. Gorleku O A, Barns A M, Prescott G R, et al. Endoplasmic reticulum localization of DHHC palmitoyltransferases mediated by lysine-based sorting signals[J]. Journal of Biological Chemistry, 2011, 286(45): 39573-39584.*

*Ref 2. Meng X, Veit M. Palmitoylation of the hemagglutinin of influenza B virus by ER-localized DHHC enzymes 1, 2, 4, and 6 is required for efficient virus replication[J]. Journal of Virology, 2023, 97(10): e01245-23.*

*Inhibition of ZDHHC6, which is directly associated with the decrease in Ki67-positive colon cancer cells. Does a functional assay support this?*

Thank you very much for your comments here. As presented below, considering that Ki67 is an antigen associated with proliferating cells, its function is closely related to mitosis and is indispensable in cell proliferation, we conducted additional experiments to specifically determine the function of ZDHHC6 in colorectal cancer (CRC). We investigated the impact of introducing Ad*ZDHHC6* or Adsh*ZDHHC6* into SNU-C2A and HCT116 cells on their rate of cell division (Supplementary figure 3A, B). Interestingly, SNU-C2A and HCT116 cells that were transfected with AdshZDHHC6 showed a significant decrease in cell viability and EdU staining intensity. Conversely, cells transfected with Ad*ZDHHC6* exhibited an increase in cell viability and EdU intensity. These findings suggest that alterations in ZDHHC6 expression in CRC cells may have an impact on proliferation in a laboratory setting (Supplementary figure 3C, D). Furthermore, our current study supports the notion that ZDHHC6 may play a role in the development of colorectal cancer (CRC) pathogenesis. This is consistent with our findings, as demonstrated by the transwell analysis, which showed a significant decrease in the invasion and migration of tumor cells when ZDHHC6 was silenced, compared to the control group (Supplementary figure 3E, F).

*However, the underlying explanation of this aberrant overexpression in CRC tissues remains uncertain. overexpression of lipids? This is unclear.*

We apologize for our inaccurate representation. According to your comments, we have corrected these statements as: “*However, the potential explanation for this abnormally vigorous lipid metabolism in CRC tissues remains uncertain.*”

*Through a bioinformatics investigation of ZDHHC6 using publicly available information, we discovered a strong correlation between the atypical expression of ZDHHC6 and worse prognosis in CRC. This sentence is a repetition (Fig. 1H). The authors should highlight the connection between ZDHHC6 and metabolism.*

Thank you very much for your constructive comments. We have deleted the part that repeated the statement, corrected the statement, and made more discussion and presentation in our revised manuscript to highlight the correlation of ZDHHC6 with lipid metabolism in CRC progression and to make our work easier to understand and read.

*Epithelial-mesenchymal transition (EMT)-related markers such as vimentin, N-cadherin.... B-catenin is a driver of CRC pathogenesis. Did the authors investigate the expression of this protein?*

Thank you very much for your comments here. We investigated the impact of introducing Ad*ZDHHC6* or Adsh*ZDHHC6* into SNU-C2A and HCT116 cell, as expected, the levels of epithelial-mesenchymal transition (EMT)-related markers such as vimentin, N-cadherin, fibronectin, TGFβ1, and MMP13 were significantly reduced in SNU-C2A and HCT116 cells. Conversely, the expression of E-cadherin was greatly increased (Supplementary figure 3G, H).

(Supplementary figure 3G, H)

Consistent with mRNA expression levels, the significant protein expression alterations of E-cadherin, N-cadherin, and TGF-beta 1 were also observed in transfected HCT116 and SNU-C2A cells (indicated below).
